# Personalized predictions of adverse side effects of the COVID-19 vaccines

**DOI:** 10.1016/j.heliyon.2022.e12753

**Published:** 2022-12-30

**Authors:** Elham Jamshidi, Amirhossein Asgary, Ali Yazdizadeh Kharrazi, Nader Tavakoli, Alireza Zali, Maryam Mehrazi, Masoud Jamshidi, Babak Farrokhi, Ali Maher, Christophe von Garnier, Sahand Jamal Rahi, Nahal Mansouri

**Affiliations:** a Functional Neurosurgery Research Center, Shohada Tajrish Comprehensive Neurosurgical Center of Excellence, Shahid Beheshti University of Medical Sciences, Tehran, Iran; b Department of Biotechnology, College of Sciences, University of Tehran, Tehran, Iran; c Trauma and Injury Research Center, Iran University of Medical Sciences, Tehran, Iran; d Department of Exercise Physiology, Tehran University, Tehran, Iran; e Health Network Administration Center, Undersecretary for Health Affairs, Ministry of Health and Medical Education, Tehran, Iran; f School of Management and Medical Education, Shahid Beheshti University of Medical Sciences, Tehran, Iran; g Division of Pulmonary Medicine, Department of Medicine, Lausanne University Hospital (CHUV), University of Lausanne (UNIL), Lausanne, Switzerland; h Laboratory of the Physics of Biological Systems, Institute of Physics, École polytechnique fédérale de Lausanne (EPFL), Lausanne, Switzerland; i Swiss Institute for Experimental Cancer Research (ISREC), School of Life Sciences, École polytechnique fédérale de Lausanne (EPFL), Lausanne, Switzerland; j Research Group on Artificial Intelligence in Pulmonary Medicine, Division of Pulmonary Medicine, Lausanne University Hospital (CHUV), Lausanne, Switzerland

**Keywords:** COVID-19, Artificial intelligence, Machine learning, Symptom, Vaccine, Adverse side effects, Sputnik V, AZD1222, AstraZeneca, Sinopharm, Moderna, Pfizer, Covaxin, AI, artificial intelligence, COVID-19, coronavirus disease of 2019, LR, logistic regression, ML, machine learning, RF, random forest, ROC, receiver operating characteristic, MLP, Multi-Layer Perceptron, KNN, K Nearest Neighbors

## Abstract

**Background:**

Misconceptions about adverse side effects are thought to influence public acceptance of the Coronavirus disease 2019 (COVID-19) vaccines negatively. To address such perceived disadvantages of vaccines, a novel machine learning (ML) approach was designed to generate personalized predictions of the most common adverse side effects following injection of six different COVID-19 vaccines based on personal and health-related characteristics.

**Methods:**

Prospective data of adverse side effects following COVID-19 vaccination in 19943 participants from Iran and Switzerland was utilized. Six vaccines were studied: The AZD1222, Sputnik V, BBIBP-CorV, COVAXIN, BNT162b2, and the mRNA-1273 vaccine. The eight side effects were considered as the model output: fever, fatigue, headache, nausea, chills, joint pain, muscle pain, and injection site reactions. The total input parameters for the first and second dose predictions were 46 and 54 features, respectively, including age, gender, lifestyle variables, and medical history. The performances of multiple ML models were compared using Area Under the Receiver Operating Characteristic Curve (ROC-AUC).

**Results:**

The total number of people receiving the first dose of the AZD1222, Sputnik V, BBIBP-CorV, COVAXIN, BNT162b2, and mRNA-1273 were 6022, 7290, 5279, 802, 277, and 273, respectively. For the second dose, the numbers were 2851, 5587, 3841, 599, 242 and 228. The Logistic Regression model for predicting different side effects of the first dose achieved ROC-AUCs of 0.620–0.686, 0.685–0.716, 0.632–0.727, 0.527–0.598, 0.548–0.655, 0.545–0.712 for the AZD1222, Sputnik V, BBIBP-CorV, COVAXIN, BNT162b2 and mRNA-1273 vaccines, respectively. The second dose models yielded ROC-AUCs of 0.777–0.867, 0.795–0.848, 0.857–0.906, 0.788–0.875, 0.683–0.850, and 0.486–0.680, respectively.

**Conclusions:**

Using a large cohort of recipients vaccinated with COVID-19 vaccines, a novel and personalized strategy was established to predict the occurrence of the most common adverse side effects with high accuracy. This technique can serve as a tool to inform COVID-19 vaccine selection and generate personalized factsheets to curb concerns about adverse side effects.

## 1 Introduction

The devastating Coronavirus disease 2019 (COVID-19) pandemic, which was initially deemed impossible to control despite numerous strategies, such as strict personal hygiene guidelines and social distancing, required establishing a global vaccination strategy [1]. The COVID-19 vaccines are one part of the solution to control the crisis. Fortunately, the steps toward using vaccination as the primary tactic against the pandemic were accelerated by the World Health Organization (WHO) Emergency Use Listing (EUL) issuance, designating the COVID-19 approved vaccines [2].

Although vaccination is essential to limit the spread of COVID-19, its success is dependent on the fact that enough individuals would be willing to get vaccinated, but some proportions of the general public show hesitancy. This vaccine hesitancy originates from various concerns, from distrusting governments and pharmaceutical companies to fearing the adverse side effects of vaccines. Vaccines are one of the most potent weapons against many infectious diseases, but at the same time, their side effects still generate intricacies among diverse populations [[3], [4], [5], [6], [7]].

COVID-19 vaccines have shown numerous adverse side effects ranging from local side effects to systemic side effects. These adverse side effects mostly include minor and mild side effects such as headache, fever and pain in the injection area[8,9]. On the other hand, some rare but concerning severe side effects such as thrombotic events and myocarditis cases have been reported [[10], [11], [12], [13]].

Vaccine adverse effects are correlated to the activity of the immune system, and the latter is closely related to sex, age, underlying disorders, and drug history [14]. In 2018, Kopsaftis Z et al. reported enhanced injection site side effects of influenza vaccines in elderly and Chronic obstructive pulmonary disease (COPD) patients [15]. Immunocompromised patients with primary immunodeficiency and hematological malignancies might be susceptible to vaccine-derived infections and stronger levels of adverse effects [16,17]. There have also been studies investigating the correlations between medical and personal characteristics and adverse side effects following the injection of COVID-19 vaccines, which have revealed a clear correlation between some aspects of vaccine recipients such as age and sex with the experienced adverse reactions [9,18].

Recognizing, anticipating, and predicting the adverse side effects of vaccines, including COVID-19 vaccines, can decrease anxiety and pave the way for the next steps toward a personalized vaccinology approach [19]. To the best of our knowledge, no study to date has addressed this critical matter for any drug or vaccine.

Based on the correlation between health-related traits and adverse side effects of vaccines, applying the medical and personal records may support a personalized estimate of each individual's adverse effects. Finding a correlation between the medical and personal characteristics and the occurrence of an adverse reaction can only be achievable through a large dataset and an enormous amount of data. Due to this reason, these predictions can only be calculated for milder adverse side effects that happen with high frequency in the population and finding a correlation between health-related traits of an individual and the rare severe adverse side effects can not be achieved. Of course, even predicting the more common adverse side effects depends on many factors.

The presence of an extensive number of parameters potentially affecting the adverse effects that one would experience makes the predictions of these side effects a complex issue. Finding correlation and building prediction models between these high numbers of parameters can be best handled with more complex methodologies such as Machine Learning (ML) and Artificial intelligence (AI) [20].

AI in healthcare has undergone meaningful progress in recent years; AI has been used as a tool for diagnosis, prognosis and risk stratification, disease screening, drug discovery, and data analysis in clinical trials [[21], [22], [23]].

Since the onset of this pandemic, AI has played a pivotal role in mitigating the impacts of COVID-19. Starting from predicting the COVID-19 dynamics, scanning for drug candidates from previously approved drugs, vaccine development, predicting the severity of COVID-19 induced infection, and even analyzing the behavioral changes towards COVID-19 vaccination [[24], [25], [26], [27], [28], [29]].

In this study using health-related characteristics and personal traits, a machine learning approach was designed to predict the potential adverse side effects after COVID-19 vaccination.

For a standardized representation of the methods and result section of this paper, a modified version of the Transparent Reporting of Multivariable Prediction Model for Individual Prognosis or Diagnosis (TRIPOD) guideline was followed [30].

## 2 Methods

### 2.1 Source of data and participants

The prospective data of the most common adverse side effects were utilized following COVID-19 vaccination in 19943 participants. Data collection was performed using a completely anonymous online survey from health care personnel at 90 hospitals in Iran and at the University Hospital of Lausanne in Switzerland. No personal information was gathered during the process to follow our anonymity strategy. The healthcare authorities informed the vaccination recipients at the vaccination centers to fill out the online survey at least 72 h following vaccination. The 72 h interval was done as some adverse reactions may appear more than 24–48 h following the vaccination.

There were no additional selection criteria outside the criteria used for the vaccine eligibility. The final database used to design the models was obtained by aggregating the data of 19943 vaccine recipients who completed the survey before Aug 25, 2021. About 33.07% of these participants had not received the second dose of their vaccine upon completing the survey; the data from these participants were only used to train the first dose models.

Overall, 6022, 7290, 5279, 802, 277, and 273 individuals received the first doses of AZD1222, Sputnik V, BBIBP-CorV, COVAXIN, BNT162b2, and mRNA-1273 vaccines, respectively. 2851, 5587, 3841, 599, 242 and 228 subjects had also received the second dose of the vaccine.

### 2.2 Outcome

The participants' adverse side effects were considered as the outputs of the prediction models. Side effects were clustered into eight most common categories: fever, fatigue, headache, nausea, chills, joint pain, muscular pain, and injection site complications (including swelling, redness, and pain).

### 2.3 Predictors

Forty-six parameters including the recipients' age, sex, blood group, smoking history, drug abuse, alcohol dependency status, BMI, comorbidities, use of specific medication, and prior COVID-19 infection status (history of COVID-19 infection, degree of severity, and symptoms) were used as the predictors for the models following the first dose of vaccines.

The prediction model for the second dose included all the 46 predictors from the first dose models, plus side effects from the first dose of vaccine as additional input data (8 parameters). The total number of predictors for the second dose models was 54. Input parameters are demonstrated in Supplementary Table 1.

The selection of variables as predictors was based on the available recorded data. All these predictors were recorded via an online survey explicitly filled by the healthcare personnel three days or more after their vaccination.

### 2.4 Missing data

Study participants completed an online survey that required an answer to all the questions. Due to the absence of missing data, there was no imputation of missing values.

### 2.5 Pre-processing

Most input and output parameters were encoded as binary variables using one-hot encoding [31]. Continuous predictors, including age and BMI, were normalized using a MinMax scaler to avoid feeding models with outlier values (for example, incorrect data entered due to unintentional mistakes while completing the form) [32].

### 2.6 Machine learning methods

To ensure that models will not be overfitted on training data and are generalizable to unseen real-world data, 20% of the data was kept as a test dataset. A 5-fold cross-validation algorithm was performed on the remaining 80% of the data [33]. For this purpose, all records were randomly split into five subsets. Four subsets were used as training data, and one subset was held for model testing as a validation set. The cross-validation process was repeated four more times, with each of the five subsets being used once as the validation data. Model performance metrics were subsequently calculated separately for each training and validation model.

To compare training and validation metrics, they need to have a similar positive and negative data points distribution; this can be achieved by splitting vaccine receivers who showed specific side effects from those who did not, and putting them into five stratified subsets, then combining them into the final five subsets. The same proportion of positive and negative distribution was maintained for every side effect in each of the final five subsets.

Several machine learning techniques were evaluated for both models: Logistic Regression (LR), Random Forest (RF), Multi-Layer Perceptron (MLP), K-Nearest Neighbors (KNN), Support Vector Machine (SVM), and Gradient Boosted Decision Trees (XGBoost) [34,35].

The Scikit-learn machine learning library was used to implement both preprocessing algorithms and models [36]. Also, the XGBoost package was used for training Gradient Boosted Decision Trees [37].

### 2.7 Model performance evaluation

For the first dose models, all of the six method types were trained for each side effect. Models’ performance in 5-fold cross-validation was evaluated using accuracy, AUC-ROC, precision, and recall [38,39].

It is important to note that the first and second dose models have been trained and validated independently. The training procedure for the second dose models was similar to the first dose, except that this time models also had access to the first dose side effect data as the input.

#### 2.7.1 Model hyperparameter tuning

A separate hyperparameter tuning analysis was run on each model and each target side effect to achieve the best possible performance for each model. GridSearchCV (RandomSearchCV for models with more parameters to tune) with a Stratified-Cross-Validation was also used for this purpose [40]. The best model configuration was selected using the mean AUC-ROC value for the validation set. It is notable that as in each iteration of hyperparameter tuning, we try to improve the performance metric on the validation dataset, and it can lead to overfitting the model on validation data. To ensure that the models are not overfitted, metrics from training, validation and unseen test sets were compared.

### 2.8 Model input-output correlations and feature importance

The LR model coefficient was used to demonstrate each predictor variable's effect on each side effect's outcome. LR calculates a probability P for each input data X with the following formula where e is the napier's constant and $\beta_{i}$ is the coefficient for feature i:

$$P\left(X\right)=\frac{1}{{1+e}^{-\left(\beta_{0}+\beta_{1}X_{1}+...+\beta_{i}X_{i}+...+\beta_{n}X_{n}\right)}}$$

Finding true correlations that represent real clinical sense requires a very large dataset, therefore this process was done only for the AZD1222, Sputnik V and BBIBP-CorV vaccines that contained a large cohort of participants.

## 3 Results

### 3.1 Participants

The median age of subjects was 43 years with an interquartile range (IQR) of 33–57.9344 subjects (46.9%) were men, and 10599 (53.1%) were women. Overall, 5639 subjects (28.28%) out of the total participants were previously infected with COVID-19.

**Fig. 1:**
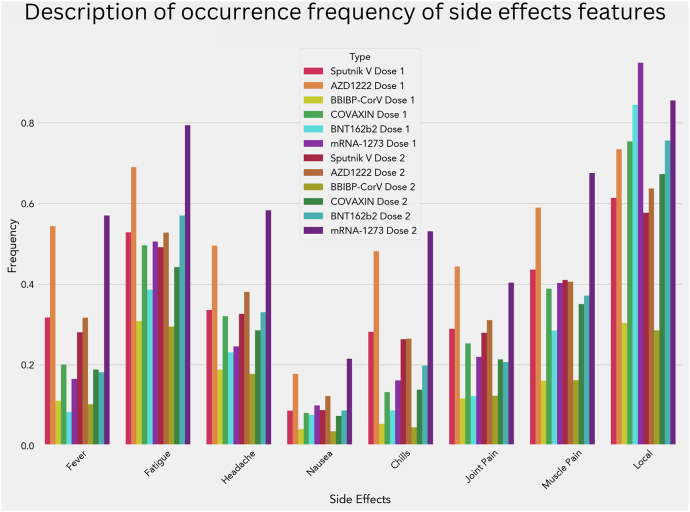
**Description of occurrence frequency of side effects features.** Description of occurrence frequency of side effects features for both doses that have been used as output data to the models.

The 46 parameters and their availability are outlined in Supplementary Table 2. The occurrence frequency of each side effect in our dataset has been shown in Fig. 1. For all 12 groups (6 vaccines, 2 doses each), local side effects such as injection site pain, redness or swelling were the most frequent (58.17%). Nausea was the least frequent side effect (10.13%). Full details of all the side effects’ frequencies are available in Supplementary Table 3.

### 3.2 Model specification

Six machine learning methods were evaluated for every dose of each vaccine (12 groups in total) which are listed with the used hyperparameters in Supplementary Table 4. The best parameter for each of the six methods has been calculated by the hyperparameter tuning using Cross-validation (the test dataset was kept unseen in this step).

### 3.3 Model performance

#### 3.3.1 First dose side effect predictions

As we need to find the models with both strong predictions and generalizability to unseen data, models should not be overfitted on training data and should have near equal performance on validation, training, and test set. As shown in Table 1, all the model types (except KNN that seem to be overfitted on training data) have comparable performance on validation sets. The average AUC of validation sets for all the side effects are 0.654, 0.650, 0.684, 0.568, 0.630 and 0.583 for the AZD1222, Sputnik V, BBIBP-CorV, COVAXIN, BNT162b2, and the mRNA-1273 vaccines respectively. These models, however, differ in their training set values. By comparing models’ performance for validation and test set, we concluded that LR had the best total performance and the least overfitting to training data.

**Table 1:** Models’ performance for the first dose of vaccines’ side effect prediction.

		Training Set						Validation Set						Test Set					
		Logistic Regression	SVM	XGBClassifier	RF	KNN	MLP	Logistic Regression	SVM	XGBClassifier	RF	KNN	MLP	Logistic Regression	SVM	XGBClassifier	RF	KNN	MLP
AZD1222	Fever	0.680	0.679	0.763	0.838	1.000	0.675	0.664	0.664	0.658	0.671	0.638	0.658	0.666	0.667	0.683	0.681	0.667	0.667
	Fatigue	0.672	0.629	0.831	0.839	1.000	0.664	0.651	0.608	0.650	0.666	0.648	0.647	0.654	0.642	0.653	0.662	0.661	0.648
	Headache	0.670	0.668	0.966	0.833	1.000	0.666	0.657	0.651	0.665	0.668	0.638	0.655	0.680	0.675	0.668	0.696	0.688	0.675
	Nausea	0.703	0.580	0.956	0.876	1.000	0.682	0.684	0.573	0.674	0.686	0.651	0.679	0.709	0.499	0.706	0.727	0.701	0.704
	Chills	0.679	0.679	0.831	0.836	1.000	0.672	0.659	0.658	0.662	0.671	0.638	0.654	0.656	0.655	0.648	0.662	0.654	0.655
	Joint Pain	0.638	0.626	0.977	0.827	1.000	0.628	0.620	0.610	0.637	0.634	0.616	0.616	0.620	0.592	0.636	0.640	0.622	0.613
	Muscle Pain	0.654	0.650	0.907	0.830	1.000	0.655	0.636	0.632	0.643	0.656	0.649	0.635	0.657	0.649	0.632	0.660	0.636	0.658
	Local side effects	0.709	0.651	0.817	0.840	1.000	0.702	0.686	0.650	0.687	0.695	0.656	0.681	0.667	0.607	0.698	0.694	0.667	0.668
Sputnik V	Fever	0.709	0.575	0.839	0.854	1.000	0.675	0.675	0.578	0.645	0.676	0.617	0.650	0.630	0.509	0.609	0.638	0.611	0.615
	Fatigue	0.688	0.648	0.937	0.849	1.000	0.681	0.667	0.629	0.656	0.673	0.639	0.663	0.666	0.512	0.654	0.677	0.630	0.662
	Headache	0.702	0.585	0.811	0.856	1.000	0.688	0.675	0.556	0.673	0.683	0.644	0.667	0.675	0.607	0.665	0.677	0.657	0.678
	Nausea	0.702	0.542	0.963	0.915	1.000	0.630	0.641	0.544	0.659	0.657	0.572	0.616	0.710	0.539	0.691	0.692	0.582	0.712
	Chills	0.692	0.579	0.727	0.839	1.000	0.646	0.632	0.558	0.653	0.647	0.556	0.621	0.655	0.518	0.614	0.663	0.604	0.628
	Joint Pain	0.709	0.577	0.768	0.859	1.000	0.680	0.670	0.552	0.666	0.675	0.612	0.655	0.675	0.537	0.632	0.671	0.618	0.671
	Muscle Pain	0.699	0.559	0.961	0.872	1.000	0.682	0.666	0.552	0.646	0.677	0.621	0.656	0.666	0.503	0.645	0.676	0.624	0.641
	Local side effects	0.743	0.725	0.825	0.862	1.000	0.738	0.727	0.712	0.710	0.726	0.687	0.723	0.737	0.716	0.735	0.738	0.681	0.734
BBIBP-CorV	Fever	0.724	0.694	0.825	0.848	1.000	0.720	0.710	0.681	0.714	0.716	0.681	0.705	0.688	0.665	0.698	0.707	0.678	0.686
	Fatigue	0.687	0.686	0.945	0.830	1.000	0.683	0.672	0.672	0.666	0.679	0.664	0.669	0.669	0.670	0.668	0.678	0.677	0.667
	Headache	0.698	0.662	0.892	0.830	1.000	0.694	0.685	0.654	0.679	0.693	0.670	0.683	0.706	0.678	0.716	0.723	0.693	0.700
	Nausea	0.731	0.543	0.893	0.862	1.000	0.699	0.702	0.532	0.691	0.706	0.651	0.686	0.728	0.528	0.731	0.726	0.698	0.721
	Chills	0.729	0.654	0.797	0.855	1.000	0.722	0.716	0.631	0.716	0.722	0.673	0.708	0.752	0.295	0.741	0.756	0.729	0.749
	Joint Pain	0.708	0.653	0.945	0.831	1.000	0.704	0.693	0.640	0.696	0.699	0.656	0.688	0.679	0.632	0.682	0.671	0.672	0.676
	Muscle Pain	0.701	0.699	0.923	0.832	1.000	0.699	0.689	0.687	0.682	0.691	0.666	0.686	0.704	0.705	0.707	0.711	0.684	0.706
	Local side effects	0.701	0.696	0.814	0.830	1.000	0.701	0.689	0.684	0.684	0.695	0.667	0.687	0.718	0.715	0.711	0.722	0.707	0.715
COVAXIN	Fever	0.703	0.677	1.000	0.960	1.000	0.657	0.584	0.550	0.615	0.626	0.608	0.572	–	–	–	–	–	–
	Fatigue	0.710	0.718	0.975	0.961	0.753	0.658	0.598	0.598	0.589	0.625	0.574	0.593	–	–	–	–	–	–
	Headache	0.676	0.694	0.978	0.970	1.000	0.650	0.578	0.546	0.622	0.603	0.607	0.572	–	–	–	–	–	–
	Nausea	0.805	0.715	1.000	0.952	1.000	0.547	0.527	0.533	0.611	0.561	0.528	0.540	–	–	–	–	–	–
	Chills	0.648	0.645	0.821	0.952	1.000	0.618	0.547	0.486	0.602	0.607	0.542	0.549	–	–	–	–	–	–
	Joint Pain	0.698	0.624	0.926	0.977	0.999	0.622	0.542	0.509	0.540	0.541	0.509	0.551	–	–	–	–	–	–
	Muscle Pain	0.693	0.659	0.948	0.865	1.000	0.627	0.549	0.556	0.545	0.558	0.539	0.554	–	–	–	–	–	–
	Local side effects	0.671	0.602	0.821	0.886	1.000	0.634	0.591	0.517	0.547	0.593	0.551	0.596	–	–	–	–	–	–
BNT162b2	Fever	0.759	0.760	0.919	0.947	0.818	0.741	0.688	0.651	0.718	0.730	0.661	0.689	–	–	–	–	–	–
	Fatigue	0.679	0.696	0.860	0.921	0.766	0.663	0.626	0.608	0.589	0.624	0.567	0.648	–	–	–	–	–	–
	Headache	0.724	0.756	0.888	0.911	1.000	0.681	0.609	0.605	0.610	0.644	0.601	0.626	–	–	–	–	–	–
	Nausea	0.821	0.857	0.661	0.979	1.000	0.748	0.669	0.494	0.645	0.657	0.665	0.656	–	–	–	–	–	–
	Chills	0.762	0.782	0.743	0.994	1.000	0.706	0.689	0.621	0.714	0.704	0.665	0.700	–	–	–	–	–	–
	Joint Pain	0.689	0.678	0.727	0.908	0.748	0.642	0.545	0.506	0.608	0.634	0.617	0.546	–	–	–	–	–	–
	Muscle Pain	0.648	0.718	1.000	0.935	0.620	0.631	0.586	0.569	0.534	0.552	0.550	0.606	–	–	–	–	–	–
	Local side effects	0.828	0.828	0.829	0.985	1.000	0.520	0.712	0.591	0.690	0.693	0.601	0.597	–	–	–	–	–	–
mRNA-1273	Fever	0.823	0.809	0.998	0.998	1.000	0.600	0.581	0.611	0.593	0.546	0.580	0.547	–	–	–	–	–	–
	Fatigue	0.773	0.748	0.814	0.978	1.000	0.653	0.548	0.553	0.600	0.572	0.561	0.586	–	–	–	–	–	–
	Headache	0.772	0.784	0.805	0.959	0.888	0.694	0.650	0.605	0.574	0.613	0.566	0.628	–	–	–	–	–	–
	Nausea	0.572	0.802	0.831	1.000	0.820	0.568	0.508	0.536	0.591	0.612	0.658	0.544	–	–	–	–	–	–
	Chills	0.904	0.834	1.000	0.993	1.000	0.666	0.655	0.703	0.558	0.589	0.529	0.600	–	–	–	–	–	–
	Joint Pain	0.722	0.692	0.899	0.995	1.000	0.477	0.584	0.559	0.615	0.584	0.549	0.530	–	–	–	–	–	–
	Muscle Pain	0.752	0.723	0.987	0.967	0.656	0.568	0.582	0.561	0.544	0.519	0.544	0.585	–	–	–	–	–	–
	Local side effects	0.817	0.737	0.787	0.957	0.749	0.659	0.630	0.587	0.593	0.572	0.529	0.569	–	–	–	–	–	–

Model average performance, calculated on training, validation, and test set using AUC-ROC parameter.

First Dose Model Performance.xlsx

#### 3.3.2 Second dose side effect predictions

As expected, the addition of first dose side effects as input features improved the model predictions for second-dose side effects (Table 2). Except for KNN that showed poor performance on the validation sets, other models showed an average AUC-ROC equal to 0.783.

**Table 2:** Different models performance for the second dose vaccines’ side effect prediction.

		Training Set						Validation Set						Test Set					
		Logistic Regression	SVM	XGBClassifier	RF	KNN	MLP	Logistic Regression	SVM	XGBClassifier	RF	KNN	MLP	Logistic Regression	SVM	XGBClassifier	RF	KNN	MLP
AZD1222	Fever	0.823	0.812	0.912	0.957	1.000	0.810	0.792	0.791	0.792	0.794	0.760	0.787	0.815	0.803	0.820	0.828	0.796	0.821
	Fatigue	0.817	0.773	0.935	0.936	1.000	0.810	0.795	0.773	0.806	0.806	0.776	0.797	0.789	0.777	0.797	0.813	0.797	0.791
	Headache	0.855	0.832	0.904	0.954	1.000	0.856	0.837	0.821	0.827	0.838	0.814	0.840	0.839	0.803	0.832	0.850	0.825	0.842
	Nausea	0.885	0.860	0.918	0.929	1.000	0.877	0.867	0.841	0.863	0.871	0.818	0.867	0.889	0.886	0.896	0.890	0.834	0.885
	Chills	0.803	0.801	0.921	0.944	1.000	0.803	0.777	0.775	0.786	0.793	0.761	0.778	0.808	0.767	0.811	0.815	0.788	0.805
	Joint Pain	0.841	0.815	0.975	0.969	1.000	0.836	0.821	0.807	0.822	0.829	0.806	0.819	0.845	0.842	0.850	0.844	0.838	0.850
	Muscle Pain	0.827	0.802	0.958	0.918	1.000	0.824	0.811	0.787	0.808	0.817	0.803	0.813	0.829	0.785	0.815	0.816	0.793	0.831
	Local side effects	0.805	0.762	0.883	0.922	1.000	0.811	0.796	0.763	0.791	0.804	0.772	0.797	0.802	0.768	0.807	0.823	0.791	0.801
Sputnik V	Fever	0.923	0.885	0.975	0.977	1.000	0.923	0.906	0.870	0.902	0.912	0.833	0.906	0.860	0.867	0.867	0.873	0.813	0.857
	Fatigue	0.907	0.883	0.959	0.966	1.000	0.912	0.903	0.874	0.890	0.903	0.858	0.902	0.890	0.873	0.881	0.895	0.872	0.887
	Headache	0.905	0.874	0.975	0.960	1.000	0.910	0.901	0.861	0.902	0.907	0.865	0.901	0.902	0.859	0.889	0.904	0.850	0.892
	Nausea	0.888	0.860	0.901	0.985	1.000	0.869	0.857	0.816	0.844	0.868	0.659	0.857	0.842	0.806	0.845	0.813	0.688	0.843
	Chills	0.889	0.838	0.994	0.990	1.000	0.882	0.861	0.808	0.841	0.859	0.728	0.860	0.794	0.769	0.774	0.793	0.682	0.759
	Joint Pain	0.908	0.856	0.916	0.967	1.000	0.901	0.887	0.832	0.879	0.892	0.825	0.889	0.895	0.832	0.874	0.892	0.826	0.909
	Muscle Pain	0.901	0.873	0.951	0.936	1.000	0.905	0.895	0.865	0.882	0.897	0.841	0.895	0.910	0.881	0.904	0.909	0.852	0.907
	Local side effects	0.899	0.874	0.960	0.955	1.000	0.910	0.894	0.874	0.893	0.901	0.869	0.896	0.893	0.841	0.881	0.892	0.875	0.888
BBIBP-CorV	Fever	0.837	0.792	0.943	0.939	1.000	0.847	0.814	0.789	0.826	0.827	0.802	0.816	0.831	0.820	0.837	0.847	0.809	0.828
	Fatigue	0.848	0.814	0.919	0.938	1.000	0.843	0.838	0.805	0.837	0.842	0.824	0.838	0.840	0.803	0.845	0.850	0.838	0.841
	Headache	0.860	0.828	0.953	0.934	1.000	0.857	0.848	0.828	0.842	0.853	0.827	0.849	0.849	0.834	0.856	0.858	0.841	0.848
	Nausea	0.844	0.794	0.989	0.969	1.000	0.841	0.819	0.777	0.815	0.830	0.780	0.826	0.829	0.810	0.836	0.853	0.802	0.823
	Chills	0.816	0.783	0.982	0.913	1.000	0.817	0.795	0.781	0.811	0.812	0.794	0.800	0.796	0.782	0.818	0.810	0.787	0.791
	Joint Pain	0.860	0.821	0.967	0.947	1.000	0.863	0.847	0.817	0.849	0.855	0.833	0.847	0.855	0.822	0.857	0.860	0.848	0.854
	Muscle Pain	0.841	0.817	0.918	0.938	1.000	0.857	0.837	0.814	0.836	0.843	0.827	0.838	0.842	0.812	0.844	0.856	0.841	0.840
	Local side effects	0.853	0.818	0.948	0.931	1.000	0.852	0.840	0.816	0.844	0.847	0.822	0.842	0.817	0.782	0.829	0.833	0.820	0.821
COVAXIN	Fever	0.917	0.884	0.854	0.973	1.000	0.863	0.844	0.826	0.828	0.849	0.810	0.834	–	–	–	–	–	–
	Fatigue	0.885	0.898	0.946	0.983	0.875	0.869	0.844	0.834	0.838	0.851	0.813	0.844	–	–	–	–	–	–
	Headache	0.874	0.879	0.972	0.993	1.000	0.855	0.842	0.817	0.836	0.850	0.811	0.834	–	–	–	–	–	–
	Nausea	0.951	0.929	0.870	0.999	0.925	0.876	0.832	0.820	0.823	0.853	0.782	0.811	–	–	–	–	–	–
	Chills	0.873	0.876	0.931	0.988	1.000	0.843	0.788	0.772	0.784	0.805	0.682	0.783	–	–	–	–	–	–
	Joint Pain	0.899	0.894	0.913	0.987	1.000	0.879	0.875	0.849	0.869	0.873	0.839	0.873	–	–	–	–	–	–
	Muscle Pain	0.888	0.888	0.974	0.955	0.879	0.867	0.850	0.842	0.842	0.847	0.817	0.847	–	–	–	–	–	–
	Local side effects	0.861	0.859	1.000	0.988	0.861	0.838	0.788	0.781	0.805	0.816	0.781	0.790	–	–	–	–	–	–
BNT162b2	Fever	0.820	0.824	0.794	0.960	0.717	0.660	0.582	0.577	0.579	0.552	0.504	0.567	–	–	–	–	–	–
	Fatigue	0.836	0.862	0.851	0.988	0.726	0.701	0.680	0.660	0.716	0.702	0.547	0.651	–	–	–	–	–	–
	Headache	0.814	0.809	0.905	0.996	0.702	0.692	0.652	0.662	0.605	0.605	0.562	0.594	–	–	–	–	–	–
	Nausea	0.875	0.809	0.874	0.978	0.724	0.670	0.675	0.655	0.616	0.636	0.553	0.615	–	–	–	–	–	–
	Chills	0.804	0.794	0.594	0.995	1.000	0.563	0.486	0.482	0.505	0.520	0.549	0.506	–	–	–	–	–	–
	Joint Pain	0.868	0.843	0.797	0.997	1.000	0.728	0.672	0.674	0.643	0.660	0.631	0.635	–	–	–	–	–	–
	Muscle Pain	0.864	0.821	0.988	0.978	0.718	0.735	0.630	0.637	0.638	0.648	0.594	0.612	–	–	–	–	–	–
	Local side effects	0.871	0.828	0.993	0.994	0.786	0.649	0.664	0.535	0.707	0.725	0.693	0.622	–	–	–	–	–	–
mRNA-1273	Fever	0.809	0.819	0.883	0.975	1.000	0.722	0.683	0.622	0.689	0.709	0.571	0.689	–	–	–	–	–	–
	Fatigue	0.792	0.796	0.827	0.954	1.000	0.752	0.717	0.711	0.763	0.762	0.657	0.701	–	–	–	–	–	–
	Headache	0.830	0.796	0.978	0.997	0.774	0.758	0.744	0.737	0.735	0.785	0.706	0.748	–	–	–	–	–	–
	Nausea	0.913	0.904	0.905	0.997	0.840	0.763	0.715	0.649	0.787	0.760	0.643	0.725	–	–	–	–	–	–
	Chills	0.806	0.827	0.857	0.999	1.000	0.702	0.721	0.662	0.648	0.698	0.615	0.690	–	–	–	–	–	–
	Joint Pain	0.814	0.855	0.979	0.979	1.000	0.791	0.734	0.684	0.785	0.774	0.708	0.731	–	–	–	–	–	–
	Muscle Pain	0.865	0.849	0.856	0.986	0.782	0.754	0.776	0.742	0.759	0.771	0.709	0.717	–	–	–	–	–	–
	Local side effects	0.900	0.879	1.000	0.982	0.842	0.835	0.850	0.815	0.771	0.824	0.773	0.810	–	–	–	–	–	–

Model average performance has been calculated on training, validation, and test set using AUC-ROC parameter.

Second Dose Model Performance.xlsx.

Here again, like first dose predictions, some models have been overfitted to training data, so LR was selected as the most efficient model. Predictions using LR achieved an AUC-ROC of over 0.90 for some side effects (Table 2). The full performance report of all the models for both doses can be found in Supplementary Table 5.

A supplementary analysis for the prediction of side effects for the second dose of the AZD1222, Sputnik V and BBIBP-CorV vaccines without including first dose side effects and solely using the original 46 parameters was also performed. The LR models achieved AUCs of 0.687, 0.651 and 0.645 for the training, validation, and test sets for all the three mentioned vaccines. This performance is similar to the prediction performance of first dose models. (Supplementary Table 6).

#### 3.3.3 Extra-validation test and generalizability

As we need to ensure that our models can be utilized on real-world data, 20% of initial data was left unseen in both training and hyperparameter optimization steps to preclude information leakage from this test set to the model. By comparing the model's performance on various sets (Tables 1 and 2), it can be concluded that our performance on the unseen test sets is comparable to the training and validation sets, which can hint at our models' generalizability. XGBoost, KNN, and RF have been overfitted on training data in both the first and second dose models.

The SVM, MLP, and LR showed average AUCs of 0.683, 0.659, and 0.716 for training sets in all different side effects for the first dose of vaccines, respectively. For the second dose models, the average training set AUCs are 0.839, 0.812, and 0.860.

### 3.4 Model input-output correlations and feature importance

To compare LR coefficients for different features, the continuous variables were first normalized to avoid undesired or upper/under-estimation of feature effects on prediction.

**Fig. 2:**
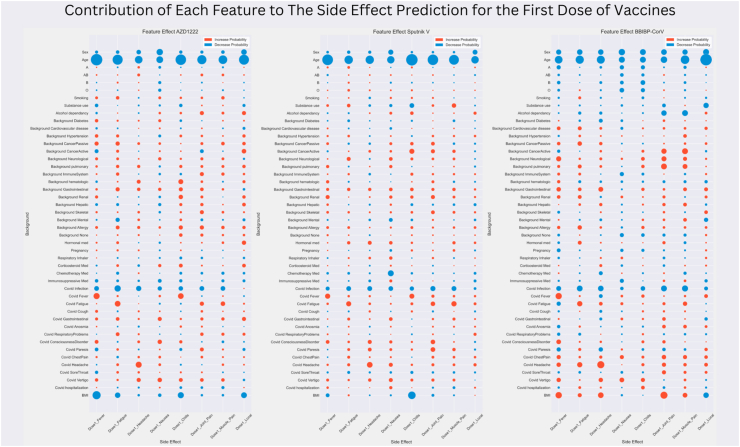
**Contribution of each feature to the side effect prediction for the first dose of vaccines.** Negative coefficients (blue circles) reflect a decreasing effect on the side effect probability. The positive coefficients (red circles) show features with an increasing effect on the probability. For the continuous variables (age and BMI) a higher value indicates a positive coefficient. Also, we encoded Male as 0 and Female as 1 in the SEX parameter.

**Fig. 3:**
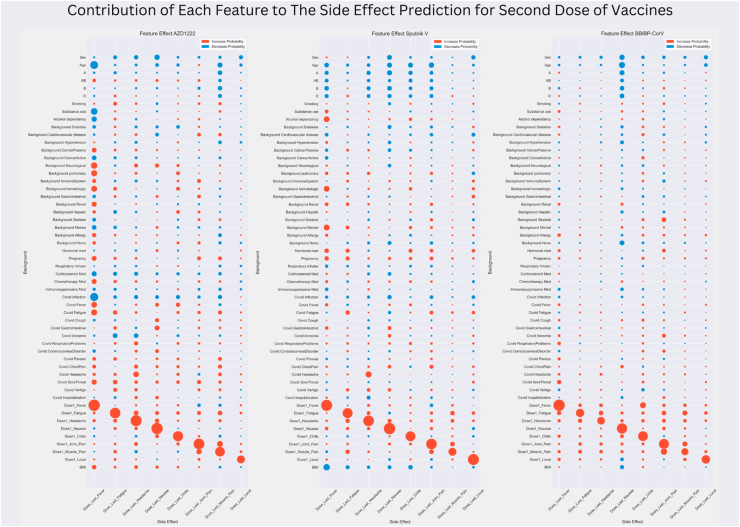
**Contribution of each feature to the side effect prediction for second dose of vaccines.** Negative coefficients (blue circles) reflect a decreasing effect on the side effect probability. The positive coefficients (red circles) show features with an increasing effect on the probability. For the continuous variables (age and BMI) a higher value indicates a positive coefficient. Also, we encoded Male as 0 and Female as 1 in the SEX parameter.

The feature importance and positive or negative correlation are shown in Figs. 2 and 3 for the first and second doses of AZD1222, Sputnik V and BBIBP-CorV vaccines. For both doses, the feature effect was similar; however, in the case of the second dose, the first dose side effects were included as additional input features. As expected, the presence of a particular side effect following the first dose has an increasing impact on their second dose counterparts.

The predictive value of input features for all three vaccines was also included separately. The detailed presentation of each input’s predictive value on all eight side effects for the three mentioned vaccines is available in Supplementary Table 7 for both the first and second dose. The five most important input features for the first dose predictions include age, previous COVID-19 infection, sex, BMI, and previous COVID-19 infection symptom (headache). For the second dose predictions, the five most important input features after excluding first dose side effects as inputs included age, sex, previous COVID-19 infection, BMI, and blood type (group A).

#### 3.4 Limited featured models

To investigate the contributions of the strongest predictors to the models’ efficiency, limited featured models were developed based on only five parameters for each dose of vaccines and side effects. The most important features for every dose and every side effect model are shown in Supplementary Table 7. The predictive values were averaged for every input parameter in the eight different side effect groups of the AZD1222, Sputnik V and BBIBP-CorV vaccines. Subsequently, the LR models were run solely based on the five most important input features as described below.

##### 3.4.1 AZD1222

The five most important features for the first dose predictions of adverse side effects include age, BMI, previous Covid-19 infection, sex, and previous Covid-19 infection symptom (vertigo). For the second dose predictions (excluding first dose features), the four main features were previous Covid-19 infection, age, previous Covid-19 infection symptoms (Sore throat). The LR models based on the five features achieved an average AUC-ROC of 0.660 (99.4% of the full-featured model) and 0.820 (99.1% of the full-featured model) for the first and second dose models respectively.

##### 3.4.2 Sputnik V

The five most important features for the first dose predictions of adverse side effects include age, previous Covid-19 infection, previous Covid-19 infection symptom (fatigue), sex and BMI. For the second dose predictions (excluding first dose features), the four main features were age, BMI, blood type group A, and blood type group B. The LR model based on the five features achieved an average AUC-ROC of 0.694 (98.3% of the full-featured model) and 0.813 (97.7% of the full-featured model) for the first and second dose models, respectively.

##### 3.4.3 BBIBP-CorV

The five most important features for the first dose predictions of adverse side effects include age, sex, previous Covid-19 infection, BMI, and previous Covid-19 infection symptom (headache). For the second dose predictions (excluding first dose features), the four main features were sex, age, allergy, and blood type group B. The LR model based on the five features achieved an average AUC-ROC of 0.669 (98.7% of the full-featured model) and 0.862 (98.6% of the full-featured model) for the first and second dose models, respectively.

## 4 Discussion

In this study, a novel machine-learning based approach was designed to predict the occurrence possibility of each common side effect for six widely approved COVID-19 vaccines solely based on recipients' personal and health-related traits. To the best of our knowledge, this is the first study to use a machine learning method to predict the occurrence of adverse side effects of any vaccine or drug based on an individual's personal and health-related characteristics.

Our findings indicate a significant correlation between the vaccine recipients’ personalized characteristics and their adverse reactions. Age had the most substantial impact on the prediction of the side effects of the first dose, which was inversely proportional to the side effects occurrence. This effect is likely due to a more robust immune response in younger individuals leading to more side effects. Interestingly, one of the other influential factors was a history of COVID-19 infection. Participants with a history of COVID-19 infection experienced more vaccine-related adverse effects. In addition, more vaccine-related adverse effects were experienced by participants with a history of cancer.

The presence of specific side effects following the first dose of vaccine substantially impacted the occurrence of that same side effect after the second dose injection. This phenomenon was also observed in previous side effects studies [41,42].

Many differences were found between genders in the presence of various side effects; Women had a higher chance of experiencing all the side effects over the 12 groups of injections in the study. This finding has been supported for other drugs as well and can be explained by a mix of factors, including inherent immune system differences among men and women and the injection dose [43].

The efficiency and acceptance of COVID-19 vaccination programs have been limited by distrust of some portions of the public [44]. Educating the public in this area can help to accelerate the speed of vaccination and establish appropriate herd immunity. The results of our study may provide support to educate the general public and provide assurance of a monitoring process of adverse events.

Since the start of this pandemic, due to uncertainties and lack of data, governments have taken decisions on vaccination programs that are potentially influenced by cognitive biases; therefore, actuating strategies replaced proficient strategies [45]. During the COVID-19 pandemic, AI has played a prominent role in tailoring fast, rapid, and cost-effective strategies and policies for policymakers against the spread of the COVID-19 pandemic [44,46]. AI-based programs are not only straightforward and accessible but also affordable and accurate.

Diverse types of machine learning methods were tested for our approach, from simple linear models (LR) to more complex models like XGBoost and Multi-Layer Perceptrons. Regarding model performance, the LR shows superior prediction performance as well as simplified and generalizable explanations that help in seeing how it decides on each outcome and how each feature affects the output.

Our study had several strengths. First, to the best of our knowledge, no other model to this date has predicted the adverse side effects of any type of vaccine based on the health-related characteristics of an individual. Second, this research is the first step toward personalized vaccinology based on side effects and can be employed for other vaccines.

With our model, personalized fact sheets can be provided to individuals for adverse side effects prior to vaccination.

However, our study had many limitations. Due to the slow speed of the vaccination process at the time of the study, this project was built on limited data for a limited number of vaccines. Increasing the dataset size may help models achieve higher generalizability to unseen data for the COVAXIN, BNT162b2, and the mRNA-1273 vaccines. Moreover, At the time of the study, the booster doses had not been rolled out, and it seems logical that the models should be extended to cover the booster vaccines for an increase in the practicality of the approach. The incapability to predict severe adverse side effects is another limitation of our model, which due to the rare occurrence of these reactions, seems unlikely to be achievable. Furthermore, the statistical significance of the input-output correlation is not as strong for the COVAXIN, BNT162b2, and the mRNA-1273 vaccines, which is mainly due to a lower number of participants for these vaccines.

In future studies, this approach can be enhanced by including more input and output data for both more practicality and more accuracy. Furthermore, this approach can easily be generalized to other vaccines and drugs and should not remain exclusive to COVID-19 vaccines. Moreover, a clinical validation study to support the real-world application of these predictions among the general public is another subject that must be studied in further investigations of this approach.

Ultimately, we anticipate that providing the public with a personalized prediction of their adverse side effects following vaccination can improve curb the general public's concerns about the COVID-19 vaccines adverse reactions. To increase the model’s applicability, a user-friendly web interface was set up (https://podsaf.org) that allows each individual to enter their own characteristics and see a prediction of their side effects following the COVID-19 vaccines.

## Author contributions

**EJ**: Conceived and designed the experiments, Analyzed and interpreted the data, Performed the experiments, Wrote the paper.

**AA**: Conceived and designed the experiments, Analyzed and interpreted the data, Performed the experiments, Wrote the paper.

**AY**: Conceived and designed the experiments, Performed the experiments, Wrote the paper.

**NT**: Conceived and designed the experiments, Performed the experiments, Wrote the paper.

**AZ**: Conceived and designed the experiments, Performed the experiments, Wrote the paper.

**AM**: Conceived and designed the experiments, Analyzed and interpreted the data, Wrote the paper.

**MJ**: Conceived and designed the experiments, Analyzed and interpreted the data.

**BF**: Conceived and designed the experiments, Analyzed and interpreted the data, Wrote the paper.

**CvG:** Performed the experiments, Analyzed and interpreted the data, Wrote the paper.

**SJ**: Conceived and designed the experiments, Analyzed and interpreted the data, Performed the experiments, Wrote the paper.

**NM**: Conceived and designed the experiments, Analyzed and interpreted the data, Performed the experiments, Wrote the paper.

## Funding

This research did not receive any specific grant from funding agencies in the public, commercial, or not-for-profit sectors.

## Data availability statement

The data that support the findings of this study are available from the below address: https://github.com/myprogrammerpersonality/vaccine-sideeffect-prediction.
