# Supplementary material for: Personalized predictions of adverse side effects of the COVID-19 vaccines
Source: Heliyon. 2022 Dec 30;9(1):e12753. doi: 10.1016/j.heliyon.2022.e12753 (PMC9800018; doi:10.1016/j.heliyon.2022.e12753)
Supplement: Multimedia component 1 [file mmc1.docx]

**Supplementary Figure .1**

A personalized fact sheet for the AZD122 vaccine based on the side effect prediction models.

**
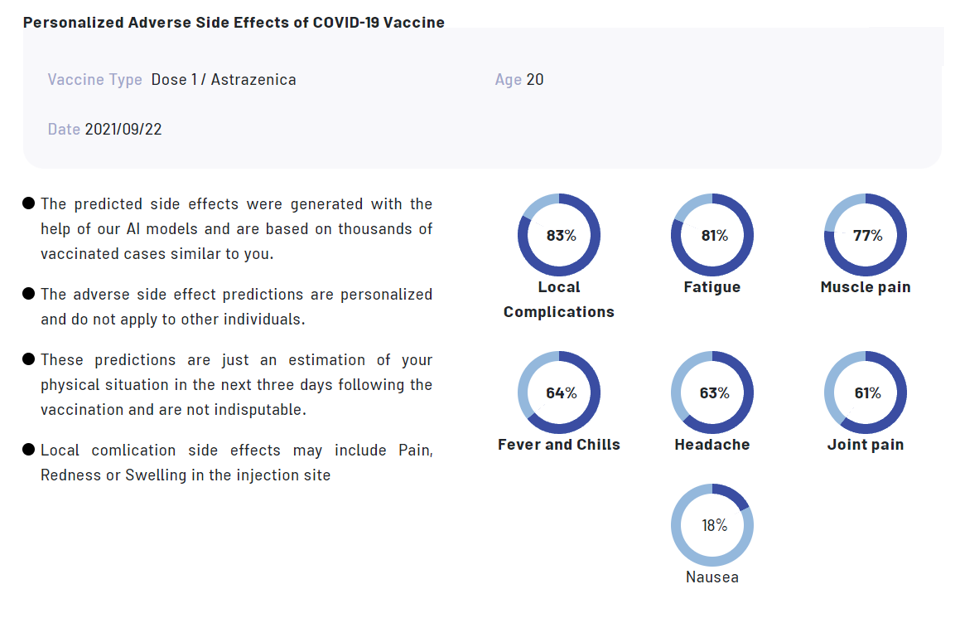
**

**Supp Table 1. Input Parameters**

Detailed list of predictor variables for the side effect prediction of COVID-19 vaccines.

| **Category** | **Name** | **Type of Paramter** |
| --- | --- | --- |
| **Age** | | **Continious** |
| **BMI** | | **Continious** |
| **Sex** | **Male** | **Binary** |
|  | **Female** |  |
| **Blood Group** | **A** | **Binary** |
|  | **AB** | **Binary** |
|  | **B** | **Binary** |
|  | **O** | **Binary** |
| **Lifestyle** | **Smoking** | **Binary** |
|  | **Substance use** | **Binary** |
|  | **Alcohol dependancy** | **Binary** |
| **Medical History** | **Background Diabetes** | **Binary** |
|  | **Background Cardiovascular disease** | **Binary** |
|  | **Background Hypertension** | **Binary** |
|  | **Background CancerPassive** | **Binary** |
|  | **Background CancerActive** | **Binary** |
|  | **Background Neurological** | **Binary** |
|  | **Background pulmonary** | **Binary** |
|  | **Background ImmuneSystem** | **Binary** |
|  | **Background hematologic** | **Binary** |
|  | **Background Gastrointestinal** | **Binary** |
|  | **Background Renal** | **Binary** |
|  | **Background Hepatic** | **Binary** |
|  | **Background Skeletal** | **Binary** |
|  | **Background Mental** | **Binary** |
|  | **Background Allergy** | **Binary** |
|  | **Background None** | **Binary** |
|  | **Hormonal med** | **Binary** |
|  | **Pregnancy** | **Binary** |
|  | **Respiratory Inhaler** | **Binary** |
|  | **Corticosteroid Med** | **Binary** |
|  | **Chemotherapy Med** | **Binary** |
|  | **Immunosuppressive Med** | **Binary** |
| **Past COVID-19 Experiences** | **Covid Infection** | **Binary** |
|  | **Covid Fever** | **Binary** |
|  | **Covid Fatigue** | **Binary** |
|  | **Covid Cough** | **Binary** |
|  | **Covid Gastrointestinal** | **Binary** |
|  | **Covid Anosmia** | **Binary** |
|  | **Covid RespiratoryProblems** | **Binary** |
|  | **Covid ConsciousnessDisorder** | **Binary** |
|  | **Covid Paresis** | **Binary** |
|  | **Covid ChestPain** | **Binary** |
|  | **Covid Headache** | **Binary** |
|  | **Covid SoreThroat** | **Binary** |
|  | **Covid Vertigo** | **Binary** |
|  | **Covid hospitalization** | **Binary** |

**Supp Table 2. Demographic Description**

Subject characteristics and their occurence in the study population.

| **Category** | **Name** | **Total Count (%)** | **Median (Q1-Q3)** | **Mean** |
| --- | --- | --- | --- | --- |
| **Age** | | 19943 | 43 (33 - 57) | 46.096 |
| **BMI** | | 19943 | 25.260 (22.857 - 28.050) | 25.757 |
|  |  | **Total Count (%)** | | |
| **Sex** | Male | 9344 (46.854) | | |
|  | Female | 10599 (53.146) | | |
| **Blood Group** | **A** | 6221 (31.194) | | |
|  | **AB** | 1932 (9.688) | | |
|  | **B** | 4378 (21.953) | | |
|  | **O** | 7338 (36.795) | | |
| **Lifestyle** | **Smoking** | 3170 (15.895) | | |
|  | **Substance use** | 235 (1.178) | | |
|  | **Alcohol dependancy** | 259 (1.299) | | |
| **Medical History** | **Background Diabetes** | 1473 (7.386) | | |
|  | **Background Cardiovascular disease** | 1433 (7.185) | | |
|  | **Background Hypertension** | 2842 (14.251) | | |
|  | **Background CancerPassive** | 553 (2.773) | | |
|  | **Background CancerActive** | 122 (0.612) | | |
|  | **Background Neurological** | 579 (2.903) | | |
|  | **Background pulmonary** | 572 (2.868) | | |
|  | **Background ImmuneSystem** | 505 (2.532) | | |
|  | **Background hematologic** | 223 (1.118) | | |
|  | **Background Gastrointestinal** | 1292 (6.478) | | |
|  | **Background Renal** | 645 (3.234) | | |
|  | **Background Hepatic** | 354 (1.775) | | |
|  | **Background Skeletal** | 1385 (6.945) | | |
|  | **Background Mental** | 305 (1.529) | | |
|  | **Background Allergy** | 2709 (13.584) | | |
|  | **Background None** | 10819 (54.25) | | |
|  | **Hormonal med** | 777 (3.896) | | |
|  | **Pregnancy** | 2741 (13.744) | | |
|  | **Respiratory Inhaler** | 3254 (16.317) | | |
|  | **Corticosteroid Med** | 2847 (14.276) | | |
|  | **Chemotherapy Med** | 2341 (11.738) | | |
|  | **Immunosuppressive Med** | 2649 (13.283) | | |
| **Past COVID-19 Experiences** | **Covid Infection** | 5639 (28.276) | | |
|  | **Covid Fever** | 3859 (19.35) | | |
|  | **Covid Fatigue** | 4700 (23.567) | | |
|  | **Covid Cough** | 2753 (13.804) | | |
|  | **Covid Gastrointestinal** | 1907 (9.562) | | |
|  | **Covid Anosmia** | 3096 (15.524) | | |
|  | **Covid RespiratoryProblems** | 1516 (7.602) | | |
|  | **Covid ConsciousnessDisorder** | 249 (1.249) | | |
|  | **Covid Paresis** | 152 (0.762) | | |
|  | **Covid ChestPain** | 1329 (6.664) | | |
|  | **Covid Headache** | 3317 (16.632) | | |
|  | **Covid SoreThroat** | 2008 (10.069) | | |
|  | **Covid Vertigo** | 1729 (8.67) | | |
|  | **Covid hospitalization** | 491 (2.462) | | |

**Supp Table 3. Dose 1 and 2 Side Effects Description**

Full details about all of the side effects’ frequencies in the study population.

| **Side Effects** | **Type** | **Dose** | **Count (%)** | **Frequency** |
| --- | --- | --- | --- | --- |
| Chills | AZD1222 | Dose 1 | 2902 (48.19) | 0.482 |
| Chills | AZD1222 | Dose 2 | 754 (26.447) | 0.264 |
| Fatigue | AZD1222 | Dose 1 | 4157 (69.03) | 0.690 |
| Fatigue | AZD1222 | Dose 2 | 1504 (52.753) | 0.528 |
| Fever | AZD1222 | Dose 1 | 3273 (54.351) | 0.544 |
| Fever | AZD1222 | Dose 2 | 903 (31.673) | 0.317 |
| Headache | AZD1222 | Dose 1 | 2981 (49.502) | 0.495 |
| Headache | AZD1222 | Dose 2 | 1086 (38.092) | 0.381 |
| Joint Pain | AZD1222 | Dose 1 | 2671 (44.354) | 0.444 |
| Joint Pain | AZD1222 | Dose 2 | 886 (31.077) | 0.311 |
| Local | AZD1222 | Dose 1 | 4421 (73.414) | 0.734 |
| Local | AZD1222 | Dose 2 | 1816 (63.697) | 0.637 |
| Muscle Pain | AZD1222 | Dose 1 | 3554 (59.017) | 0.590 |
| Muscle Pain | AZD1222 | Dose 2 | 1157 (40.582) | 0.406 |
| Nausea | AZD1222 | Dose 1 | 1067 (17.718) | 0.177 |
| Nausea | AZD1222 | Dose 2 | 349 (12.241) | 0.122 |
| Chills | BBIBP-CorV | Dose 1 | 283 (5.361) | 0.054 |
| Chills | BBIBP-CorV | Dose 2 | 174 (4.53) | 0.045 |
| Fatigue | BBIBP-CorV | Dose 1 | 1627 (30.82) | 0.308 |
| Fatigue | BBIBP-CorV | Dose 2 | 1131 (29.445) | 0.294 |
| Fever | BBIBP-CorV | Dose 1 | 585 (11.082) | 0.111 |
| Fever | BBIBP-CorV | Dose 2 | 394 (10.258) | 0.103 |
| Headache | BBIBP-CorV | Dose 1 | 993 (18.81) | 0.188 |
| Headache | BBIBP-CorV | Dose 2 | 681 (17.73) | 0.177 |
| Joint Pain | BBIBP-CorV | Dose 1 | 615 (11.65) | 0.116 |
| Joint Pain | BBIBP-CorV | Dose 2 | 473 (12.315) | 0.123 |
| Local | BBIBP-CorV | Dose 1 | 1605 (30.403) | 0.304 |
| Local | BBIBP-CorV | Dose 2 | 1096 (28.534) | 0.285 |
| Muscle Pain | BBIBP-CorV | Dose 1 | 848 (16.064) | 0.161 |
| Muscle Pain | BBIBP-CorV | Dose 2 | 621 (16.168) | 0.162 |
| Nausea | BBIBP-CorV | Dose 1 | 214 (4.054) | 0.041 |
| Nausea | BBIBP-CorV | Dose 2 | 136 (3.541) | 0.035 |
| Chills | BNT162b2 | Dose 1 | 24 (8.664) | 0.087 |
| Chills | BNT162b2 | Dose 2 | 48 (19.835) | 0.198 |
| Fatigue | BNT162b2 | Dose 1 | 107 (38.628) | 0.386 |
| Fatigue | BNT162b2 | Dose 2 | 138 (57.025) | 0.570 |
| Fever | BNT162b2 | Dose 1 | 23 (8.303) | 0.083 |
| Fever | BNT162b2 | Dose 2 | 44 (18.182) | 0.182 |
| Headache | BNT162b2 | Dose 1 | 64 (23.105) | 0.231 |
| Headache | BNT162b2 | Dose 2 | 80 (33.058) | 0.331 |
| Joint Pain | BNT162b2 | Dose 1 | 34 (12.274) | 0.123 |
| Joint Pain | BNT162b2 | Dose 2 | 50 (20.661) | 0.207 |
| Local | BNT162b2 | Dose 1 | 234 (84.477) | 0.845 |
| Local | BNT162b2 | Dose 2 | 183 (75.62) | 0.756 |
| Muscle Pain | BNT162b2 | Dose 1 | 79 (28.52) | 0.285 |
| Muscle Pain | BNT162b2 | Dose 2 | 90 (37.19) | 0.372 |
| Nausea | BNT162b2 | Dose 1 | 21 (7.581) | 0.076 |
| Nausea | BNT162b2 | Dose 2 | 21 (8.678) | 0.087 |
| Chills | COVAXIN | Dose 1 | 106 (13.217) | 0.132 |
| Chills | COVAXIN | Dose 2 | 83 (13.856) | 0.139 |
| Fatigue | COVAXIN | Dose 1 | 398 (49.626) | 0.496 |
| Fatigue | COVAXIN | Dose 2 | 265 (44.24) | 0.442 |
| Fever | COVAXIN | Dose 1 | 161 (20.075) | 0.201 |
| Fever | COVAXIN | Dose 2 | 113 (18.865) | 0.189 |
| Headache | COVAXIN | Dose 1 | 257 (32.045) | 0.320 |
| Headache | COVAXIN | Dose 2 | 171 (28.548) | 0.285 |
| Joint Pain | COVAXIN | Dose 1 | 203 (25.312) | 0.253 |
| Joint Pain | COVAXIN | Dose 2 | 128 (21.369) | 0.214 |
| Local | COVAXIN | Dose 1 | 605 (75.436) | 0.754 |
| Local | COVAXIN | Dose 2 | 403 (67.279) | 0.673 |
| Muscle Pain | COVAXIN | Dose 1 | 312 (38.903) | 0.389 |
| Muscle Pain | COVAXIN | Dose 2 | 210 (35.058) | 0.351 |
| Nausea | COVAXIN | Dose 1 | 65 (8.105) | 0.081 |
| Nausea | COVAXIN | Dose 2 | 44 (7.346) | 0.073 |
| Chills | mRNA-1273 | Dose 1 | 44 (16.117) | 0.161 |
| Chills | mRNA-1273 | Dose 2 | 121 (53.07) | 0.531 |
| Fatigue | mRNA-1273 | Dose 1 | 138 (50.549) | 0.505 |
| Fatigue | mRNA-1273 | Dose 2 | 181 (79.386) | 0.794 |
| Fever | mRNA-1273 | Dose 1 | 45 (16.484) | 0.165 |
| Fever | mRNA-1273 | Dose 2 | 130 (57.018) | 0.570 |
| Headache | mRNA-1273 | Dose 1 | 67 (24.542) | 0.245 |
| Headache | mRNA-1273 | Dose 2 | 133 (58.333) | 0.583 |
| Joint Pain | mRNA-1273 | Dose 1 | 60 (21.978) | 0.220 |
| Joint Pain | mRNA-1273 | Dose 2 | 92 (40.351) | 0.404 |
| Local | mRNA-1273 | Dose 1 | 259 (94.872) | 0.949 |
| Local | mRNA-1273 | Dose 2 | 195 (85.526) | 0.855 |
| Muscle Pain | mRNA-1273 | Dose 1 | 110 (40.293) | 0.403 |
| Muscle Pain | mRNA-1273 | Dose 2 | 154 (67.544) | 0.675 |
| Nausea | mRNA-1273 | Dose 1 | 27 (9.89) | 0.099 |
| Nausea | mRNA-1273 | Dose 2 | 49 (21.491) | 0.215 |
| Chills | Sputnik V | Dose 1 | 2055 (28.189) | 0.282 |
| Chills | Sputnik V | Dose 2 | 1472 (26.347) | 0.263 |
| Fatigue | Sputnik V | Dose 1 | 3854 (52.867) | 0.529 |
| Fatigue | Sputnik V | Dose 2 | 2747 (49.168) | 0.492 |
| Fever | Sputnik V | Dose 1 | 2313 (31.728) | 0.317 |
| Fever | Sputnik V | Dose 2 | 1569 (28.083) | 0.281 |
| Headache | Sputnik V | Dose 1 | 2448 (33.58) | 0.336 |
| Headache | Sputnik V | Dose 2 | 1822 (32.611) | 0.326 |
| Joint Pain | Sputnik V | Dose 1 | 2108 (28.916) | 0.289 |
| Joint Pain | Sputnik V | Dose 2 | 1561 (27.94) | 0.279 |
| Local | Sputnik V | Dose 1 | 4477 (61.413) | 0.614 |
| Local | Sputnik V | Dose 2 | 3225 (57.723) | 0.577 |
| Muscle Pain | Sputnik V | Dose 1 | 3179 (43.608) | 0.436 |
| Muscle Pain | Sputnik V | Dose 2 | 2291 (41.006) | 0.410 |
| Nausea | Sputnik V | Dose 1 | 628 (8.615) | 0.086 |
| Nausea | Sputnik V | Dose 2 | 488 (8.735) | 0.087 |

**Supp Table 4. Hyperparameters of Models:**

Hyperparameters of all the six machine learning methods for every dose of each vaccine.

|  | **SideEffect** | **ModelType** | **Param** |
| --- | --- | --- | --- |
| **1** | Dose1_Fever | Logistic_Regression | C: 1.0, solver: lbfgs |
| **2** | Dose1_Fatigue | Logistic_Regression | C: 0.5, solver: lbfgs |
| **3** | Dose1_Headache | Logistic_Regression | C: 0.1, solver: liblinear |
| **4** | Dose1_Nausea | Logistic_Regression | C: 0.1, solver: liblinear |
| **5** | Dose1_Chills | Logistic_Regression | C: 0.5, solver: lbfgs |
| **6** | Dose1_Joint_Pain | Logistic_Regression | C: 0.5, solver: liblinear |
| **7** | Dose1_Muscle_Pain | Logistic_Regression | C: 0.1, solver: lbfgs |
| **8** | Dose1_Local | Logistic_Regression | C: 0.5, solver: lbfgs |
| **9** | Dose1_Fever | SVM | C: 10, gamma: scale, kernel: linear |
| **10** | Dose1_Fatigue | SVM | C: 1, gamma: scale, kernel: linear |
| **11** | Dose1_Headache | SVM | C: 10, gamma: scale, kernel: linear |
| **12** | Dose1_Nausea | SVM | C: 10, gamma: scale, kernel: linear |
| **13** | Dose1_Chills | SVM | C: 1, gamma: scale, kernel: linear |
| **14** | Dose1_Joint_Pain | SVM | C: 1, gamma: scale, kernel: linear |
| **15** | Dose1_Muscle_Pain | SVM | C: 10, gamma: scale, kernel: linear |
| **16** | Dose1_Local | SVM | C: 0.1, gamma: scale, kernel: linear |
| **17** | Dose1_Fever | XGBClassifier | n_estimators: 150, min_child_weight: 3, max_depth: 6, learning_rate: 0.01 |
| **18** | Dose1_Fatigue | XGBClassifier | n_estimators: 50, min_child_weight: 4, max_depth: 8, learning_rate: 0.1 |
| **19** | Dose1_Headache | XGBClassifier | n_estimators: 100, min_child_weight: 2, max_depth: 9, learning_rate: 0.1 |
| **20** | Dose1_Nausea | XGBClassifier | n_estimators: 150, min_child_weight: 3, max_depth: 10, learning_rate: 0.1 |
| **21** | Dose1_Chills | XGBClassifier | n_estimators: 50, min_child_weight: 1, max_depth: 8, learning_rate: 0.1 |
| **22** | Dose1_Joint_Pain | XGBClassifier | n_estimators: 50, min_child_weight: 3, max_depth: 9, learning_rate: 0.1 |
| **23** | Dose1_Muscle_Pain | XGBClassifier | n_estimators: 50, min_child_weight: 3, max_depth: 10, learning_rate: 0.1 |
| **24** | Dose1_Local | XGBClassifier | n_estimators: 50, min_child_weight: 4, max_depth: 6, learning_rate: 0.1 |
| **25** | Dose1_Fever | RF | max_depth: 9, min_samples_split: 2, n_estimators: 50 |
| **26** | Dose1_Fatigue | RF | max_depth: 10, min_samples_split: 3, n_estimators: 150 |
| **27** | Dose1_Headache | RF | max_depth: 10, min_samples_split: 2, n_estimators: 150 |
| **28** | Dose1_Nausea | RF | max_depth: 10, min_samples_split: 3, n_estimators: 50 |
| **29** | Dose1_Chills | RF | max_depth: 10, min_samples_split: 3, n_estimators: 100 |
| **30** | Dose1_Joint_Pain | RF | max_depth: 10, min_samples_split: 2, n_estimators: 50 |
| **31** | Dose1_Muscle_Pain | RF | max_depth: 10, min_samples_split: 4, n_estimators: 50 |
| **32** | Dose1_Local | RF | max_depth: 10, min_samples_split: 4, n_estimators: 100 |
| **33** | Dose1_Fever | KNN | n_neighbors: 13, weights: distance |
| **34** | Dose1_Fatigue | KNN | n_neighbors: 13, weights: distance |
| **35** | Dose1_Headache | KNN | n_neighbors: 13, weights: distance |
| **36** | Dose1_Nausea | KNN | n_neighbors: 13, weights: distance |
| **37** | Dose1_Chills | KNN | n_neighbors: 13, weights: distance |
| **38** | Dose1_Joint_Pain | KNN | n_neighbors: 13, weights: distance |
| **39** | Dose1_Muscle_Pain | KNN | n_neighbors: 9, weights: distance |
| **40** | Dose1_Local | KNN | n_neighbors: 13, weights: distance |
| **41** | Dose1_Fever | MLP | activation: tanh, hidden_layer_sizes: (100, 100), learning_rate: adaptive, solver: sgd |
| **42** | Dose1_Fatigue | MLP | activation: tanh, hidden_layer_sizes: (100, 100), learning_rate: constant, solver: sgd |
| **43** | Dose1_Headache | MLP | activation: tanh, hidden_layer_sizes: (100, 100), learning_rate: constant, solver: sgd |
| **44** | Dose1_Nausea | MLP | activation: tanh, hidden_layer_sizes: (100, 100), learning_rate: adaptive, solver: sgd |
| **45** | Dose1_Chills | MLP | activation: tanh, hidden_layer_sizes: (100, 100), learning_rate: constant, solver: sgd |
| **46** | Dose1_Joint_Pain | MLP | activation: tanh, hidden_layer_sizes: (100, 100), learning_rate: adaptive, solver: sgd |
| **47** | Dose1_Muscle_Pain | MLP | activation: tanh, hidden_layer_sizes: (100, 100), learning_rate: adaptive, solver: sgd |
| **48** | Dose1_Local | MLP | activation: tanh, hidden_layer_sizes: (100, 100), learning_rate: constant, solver: sgd |

**Supp Table 5. Full Performance Report for Models:**

The full performance report of all the models for side effect predictions.

|  |  | Training Set | | | | | | Validation Set | | | | | |
| --- | --- | --- | --- | --- | --- | --- | --- | --- | --- | --- | --- | --- | --- |
|  |  | Logistic Regression | SVM | XGBClassifier | RF | KNN | MLP | Logistic Regression | SVM | XGBClassifier | RF | KNN | MLP |
| AZD1222 | Fever | 0.680 | 0.679 | 0.763 | 0.838 | 1.000 | 0.675 | 0.664 | 0.664 | 0.658 | 0.671 | 0.638 | 0.658 |
|  | Fatigue | 0.672 | 0.629 | 0.831 | 0.839 | 1.000 | 0.664 | 0.651 | 0.608 | 0.650 | 0.666 | 0.648 | 0.647 |
|  | Headache | 0.670 | 0.668 | 0.966 | 0.833 | 1.000 | 0.666 | 0.657 | 0.651 | 0.665 | 0.668 | 0.638 | 0.655 |
|  | Nausea | 0.703 | 0.580 | 0.956 | 0.876 | 1.000 | 0.682 | 0.684 | 0.573 | 0.674 | 0.686 | 0.651 | 0.679 |
|  | Chills | 0.679 | 0.679 | 0.831 | 0.836 | 1.000 | 0.672 | 0.659 | 0.658 | 0.662 | 0.671 | 0.638 | 0.654 |
|  | Joint Pain | 0.638 | 0.626 | 0.977 | 0.827 | 1.000 | 0.628 | 0.620 | 0.610 | 0.637 | 0.634 | 0.616 | 0.616 |
|  | Muscle Pain | 0.654 | 0.650 | 0.907 | 0.830 | 1.000 | 0.655 | 0.636 | 0.632 | 0.643 | 0.656 | 0.649 | 0.635 |
|  | Local side effects | 0.709 | 0.651 | 0.817 | 0.840 | 1.000 | 0.702 | 0.686 | 0.650 | 0.687 | 0.695 | 0.656 | 0.681 |
| Sputnik V | Fever | 0.709 | 0.575 | 0.839 | 0.854 | 1.000 | 0.675 | 0.675 | 0.578 | 0.645 | 0.676 | 0.617 | 0.650 |
|  | Fatigue | 0.688 | 0.648 | 0.937 | 0.849 | 1.000 | 0.681 | 0.667 | 0.629 | 0.656 | 0.673 | 0.639 | 0.663 |
|  | Headache | 0.702 | 0.585 | 0.811 | 0.856 | 1.000 | 0.688 | 0.675 | 0.556 | 0.673 | 0.683 | 0.644 | 0.667 |
|  | Nausea | 0.702 | 0.542 | 0.963 | 0.915 | 1.000 | 0.630 | 0.641 | 0.544 | 0.659 | 0.657 | 0.572 | 0.616 |
|  | Chills | 0.692 | 0.579 | 0.727 | 0.839 | 1.000 | 0.646 | 0.632 | 0.558 | 0.653 | 0.647 | 0.556 | 0.621 |
|  | Joint Pain | 0.709 | 0.577 | 0.768 | 0.859 | 1.000 | 0.680 | 0.670 | 0.552 | 0.666 | 0.675 | 0.612 | 0.655 |
|  | Muscle Pain | 0.699 | 0.559 | 0.961 | 0.872 | 1.000 | 0.682 | 0.666 | 0.552 | 0.646 | 0.677 | 0.621 | 0.656 |
|  | Local side effects | 0.743 | 0.725 | 0.825 | 0.862 | 1.000 | 0.738 | 0.727 | 0.712 | 0.710 | 0.726 | 0.687 | 0.723 |
| BBIBP-CorV | Fever | 0.724 | 0.694 | 0.825 | 0.848 | 1.000 | 0.720 | 0.710 | 0.681 | 0.714 | 0.716 | 0.681 | 0.705 |
|  | Fatigue | 0.687 | 0.686 | 0.945 | 0.830 | 1.000 | 0.683 | 0.672 | 0.672 | 0.666 | 0.679 | 0.664 | 0.669 |
|  | Headache | 0.698 | 0.662 | 0.892 | 0.830 | 1.000 | 0.694 | 0.685 | 0.654 | 0.679 | 0.693 | 0.670 | 0.683 |
|  | Nausea | 0.731 | 0.543 | 0.893 | 0.862 | 1.000 | 0.699 | 0.702 | 0.532 | 0.691 | 0.706 | 0.651 | 0.686 |
|  | Chills | 0.729 | 0.654 | 0.797 | 0.855 | 1.000 | 0.722 | 0.716 | 0.631 | 0.716 | 0.722 | 0.673 | 0.708 |
|  | Joint Pain | 0.708 | 0.653 | 0.945 | 0.831 | 1.000 | 0.704 | 0.693 | 0.640 | 0.696 | 0.699 | 0.656 | 0.688 |
|  | Muscle Pain | 0.701 | 0.699 | 0.923 | 0.832 | 1.000 | 0.699 | 0.689 | 0.687 | 0.682 | 0.691 | 0.666 | 0.686 |
|  | Local side effects | 0.701 | 0.696 | 0.814 | 0.830 | 1.000 | 0.701 | 0.689 | 0.684 | 0.684 | 0.695 | 0.667 | 0.687 |
| COVAXIN | Fever | 0.703 | 0.677 | 1.000 | 0.960 | 1.000 | 0.657 | 0.584 | 0.550 | 0.615 | 0.626 | 0.608 | 0.572 |
|  | Fatigue | 0.710 | 0.718 | 0.975 | 0.961 | 0.753 | 0.658 | 0.598 | 0.598 | 0.589 | 0.625 | 0.574 | 0.593 |
|  | Headache | 0.676 | 0.694 | 0.978 | 0.970 | 1.000 | 0.650 | 0.578 | 0.546 | 0.622 | 0.603 | 0.607 | 0.572 |
|  | Nausea | 0.805 | 0.715 | 1.000 | 0.952 | 1.000 | 0.547 | 0.527 | 0.533 | 0.611 | 0.561 | 0.528 | 0.540 |
|  | Chills | 0.648 | 0.645 | 0.821 | 0.952 | 1.000 | 0.618 | 0.547 | 0.486 | 0.602 | 0.607 | 0.542 | 0.549 |
|  | Joint Pain | 0.698 | 0.624 | 0.926 | 0.977 | 0.999 | 0.622 | 0.542 | 0.509 | 0.540 | 0.541 | 0.509 | 0.551 |
|  | Muscle Pain | 0.693 | 0.659 | 0.948 | 0.865 | 1.000 | 0.627 | 0.549 | 0.556 | 0.545 | 0.558 | 0.539 | 0.554 |
|  | Local side effects | 0.671 | 0.602 | 0.821 | 0.886 | 1.000 | 0.634 | 0.591 | 0.517 | 0.547 | 0.593 | 0.551 | 0.596 |
| BNT162b2 | Fever | 0.759 | 0.760 | 0.919 | 0.947 | 0.818 | 0.741 | 0.688 | 0.651 | 0.718 | 0.730 | 0.661 | 0.689 |
|  | Fatigue | 0.679 | 0.696 | 0.860 | 0.921 | 0.766 | 0.663 | 0.626 | 0.608 | 0.589 | 0.624 | 0.567 | 0.648 |
|  | Headache | 0.724 | 0.756 | 0.888 | 0.911 | 1.000 | 0.681 | 0.609 | 0.605 | 0.610 | 0.644 | 0.601 | 0.626 |
|  | Nausea | 0.821 | 0.857 | 0.661 | 0.979 | 1.000 | 0.748 | 0.669 | 0.494 | 0.645 | 0.657 | 0.665 | 0.656 |
|  | Chills | 0.762 | 0.782 | 0.743 | 0.994 | 1.000 | 0.706 | 0.689 | 0.621 | 0.714 | 0.704 | 0.665 | 0.700 |
|  | Joint Pain | 0.689 | 0.678 | 0.727 | 0.908 | 0.748 | 0.642 | 0.545 | 0.506 | 0.608 | 0.634 | 0.617 | 0.546 |
|  | Muscle Pain | 0.648 | 0.718 | 1.000 | 0.935 | 0.620 | 0.631 | 0.586 | 0.569 | 0.534 | 0.552 | 0.550 | 0.606 |
|  | Local side effects | 0.828 | 0.828 | 0.829 | 0.985 | 1.000 | 0.520 | 0.712 | 0.591 | 0.690 | 0.693 | 0.601 | 0.597 |
| mRNA-1273 | Fever | 0.823 | 0.809 | 0.998 | 0.998 | 1.000 | 0.600 | 0.581 | 0.611 | 0.593 | 0.546 | 0.580 | 0.547 |
|  | Fatigue | 0.773 | 0.748 | 0.814 | 0.978 | 1.000 | 0.653 | 0.548 | 0.553 | 0.600 | 0.572 | 0.561 | 0.586 |
|  | Headache | 0.772 | 0.784 | 0.805 | 0.959 | 0.888 | 0.694 | 0.650 | 0.605 | 0.574 | 0.613 | 0.566 | 0.628 |
|  | Nausea | 0.572 | 0.802 | 0.831 | 1.000 | 0.820 | 0.568 | 0.508 | 0.536 | 0.591 | 0.612 | 0.658 | 0.544 |
|  | Chills | 0.904 | 0.834 | 1.000 | 0.993 | 1.000 | 0.666 | 0.655 | 0.703 | 0.558 | 0.589 | 0.529 | 0.600 |
|  | Joint Pain | 0.722 | 0.692 | 0.899 | 0.995 | 1.000 | 0.477 | 0.584 | 0.559 | 0.615 | 0.584 | 0.549 | 0.530 |
|  | Muscle Pain | 0.752 | 0.723 | 0.987 | 0.967 | 0.656 | 0.568 | 0.582 | 0.561 | 0.544 | 0.519 | 0.544 | 0.585 |
|  | Local side effects | 0.817 | 0.737 | 0.787 | 0.957 | 0.749 | 0.659 | 0.630 | 0.587 | 0.593 | 0.572 | 0.529 | 0.569 |
| AZD1222 - Dose 2 | Fever | 0.823 | 0.812 | 0.912 | 0.957 | 1.000 | 0.810 | 0.792 | 0.791 | 0.792 | 0.794 | 0.760 | 0.787 |
|  | Fatigue | 0.817 | 0.773 | 0.935 | 0.936 | 1.000 | 0.810 | 0.795 | 0.773 | 0.806 | 0.806 | 0.776 | 0.797 |
|  | Headache | 0.855 | 0.832 | 0.904 | 0.954 | 1.000 | 0.856 | 0.837 | 0.821 | 0.827 | 0.838 | 0.814 | 0.840 |
|  | Nausea | 0.885 | 0.860 | 0.918 | 0.929 | 1.000 | 0.877 | 0.867 | 0.841 | 0.863 | 0.871 | 0.818 | 0.867 |
|  | Chills | 0.803 | 0.801 | 0.921 | 0.944 | 1.000 | 0.803 | 0.777 | 0.775 | 0.786 | 0.793 | 0.761 | 0.778 |
|  | Joint Pain | 0.841 | 0.815 | 0.975 | 0.969 | 1.000 | 0.836 | 0.821 | 0.807 | 0.822 | 0.829 | 0.806 | 0.819 |
|  | Muscle Pain | 0.827 | 0.802 | 0.958 | 0.918 | 1.000 | 0.824 | 0.811 | 0.787 | 0.808 | 0.817 | 0.803 | 0.813 |
|  | Local side effects | 0.805 | 0.762 | 0.883 | 0.922 | 1.000 | 0.811 | 0.796 | 0.763 | 0.791 | 0.804 | 0.772 | 0.797 |
| Sputnik V - Dose 2 | Fever | 0.923 | 0.885 | 0.975 | 0.977 | 1.000 | 0.923 | 0.906 | 0.870 | 0.902 | 0.912 | 0.833 | 0.906 |
|  | Fatigue | 0.907 | 0.883 | 0.959 | 0.966 | 1.000 | 0.912 | 0.903 | 0.874 | 0.890 | 0.903 | 0.858 | 0.902 |
|  | Headache | 0.905 | 0.874 | 0.975 | 0.960 | 1.000 | 0.910 | 0.901 | 0.861 | 0.902 | 0.907 | 0.865 | 0.901 |
|  | Nausea | 0.888 | 0.860 | 0.901 | 0.985 | 1.000 | 0.869 | 0.857 | 0.816 | 0.844 | 0.868 | 0.659 | 0.857 |
|  | Chills | 0.889 | 0.838 | 0.994 | 0.990 | 1.000 | 0.882 | 0.861 | 0.808 | 0.841 | 0.859 | 0.728 | 0.860 |
|  | Joint Pain | 0.908 | 0.856 | 0.916 | 0.967 | 1.000 | 0.901 | 0.887 | 0.832 | 0.879 | 0.892 | 0.825 | 0.889 |
|  | Muscle Pain | 0.901 | 0.873 | 0.951 | 0.936 | 1.000 | 0.905 | 0.895 | 0.865 | 0.882 | 0.897 | 0.841 | 0.895 |
|  | Local side effects | 0.899 | 0.874 | 0.960 | 0.955 | 1.000 | 0.910 | 0.894 | 0.874 | 0.893 | 0.901 | 0.869 | 0.896 |
| BBIBP-CorV - Dose 2 | Fever | 0.837 | 0.792 | 0.943 | 0.939 | 1.000 | 0.847 | 0.814 | 0.789 | 0.826 | 0.827 | 0.802 | 0.816 |
|  | Fatigue | 0.848 | 0.814 | 0.919 | 0.938 | 1.000 | 0.843 | 0.838 | 0.805 | 0.837 | 0.842 | 0.824 | 0.838 |
|  | Headache | 0.860 | 0.828 | 0.953 | 0.934 | 1.000 | 0.857 | 0.848 | 0.828 | 0.842 | 0.853 | 0.827 | 0.849 |
|  | Nausea | 0.844 | 0.794 | 0.989 | 0.969 | 1.000 | 0.841 | 0.819 | 0.777 | 0.815 | 0.830 | 0.780 | 0.826 |
|  | Chills | 0.816 | 0.783 | 0.982 | 0.913 | 1.000 | 0.817 | 0.795 | 0.781 | 0.811 | 0.812 | 0.794 | 0.800 |
|  | Joint Pain | 0.860 | 0.821 | 0.967 | 0.947 | 1.000 | 0.863 | 0.847 | 0.817 | 0.849 | 0.855 | 0.833 | 0.847 |
|  | Muscle Pain | 0.841 | 0.817 | 0.918 | 0.938 | 1.000 | 0.857 | 0.837 | 0.814 | 0.836 | 0.843 | 0.827 | 0.838 |
|  | Local side effects | 0.853 | 0.818 | 0.948 | 0.931 | 1.000 | 0.852 | 0.840 | 0.816 | 0.844 | 0.847 | 0.822 | 0.842 |
| COVAXIN - Dose 2 | Fever | 0.917 | 0.884 | 0.854 | 0.973 | 1.000 | 0.863 | 0.844 | 0.826 | 0.828 | 0.849 | 0.810 | 0.834 |
|  | Fatigue | 0.885 | 0.898 | 0.946 | 0.983 | 0.875 | 0.869 | 0.844 | 0.834 | 0.838 | 0.851 | 0.813 | 0.844 |
|  | Headache | 0.874 | 0.879 | 0.972 | 0.993 | 1.000 | 0.855 | 0.842 | 0.817 | 0.836 | 0.850 | 0.811 | 0.834 |
|  | Nausea | 0.951 | 0.929 | 0.870 | 0.999 | 0.925 | 0.876 | 0.832 | 0.820 | 0.823 | 0.853 | 0.782 | 0.811 |
|  | Chills | 0.873 | 0.876 | 0.931 | 0.988 | 1.000 | 0.843 | 0.788 | 0.772 | 0.784 | 0.805 | 0.682 | 0.783 |
|  | Joint Pain | 0.899 | 0.894 | 0.913 | 0.987 | 1.000 | 0.879 | 0.875 | 0.849 | 0.869 | 0.873 | 0.839 | 0.873 |
|  | Muscle Pain | 0.888 | 0.888 | 0.974 | 0.955 | 0.879 | 0.867 | 0.850 | 0.842 | 0.842 | 0.847 | 0.817 | 0.847 |
|  | Local side effects | 0.861 | 0.859 | 1.000 | 0.988 | 0.861 | 0.838 | 0.788 | 0.781 | 0.805 | 0.816 | 0.781 | 0.790 |
| BNT162b2 - Dose 2 | Fever | 0.820 | 0.824 | 0.794 | 0.960 | 0.717 | 0.660 | 0.582 | 0.577 | 0.579 | 0.552 | 0.504 | 0.567 |
|  | Fatigue | 0.836 | 0.862 | 0.851 | 0.988 | 0.726 | 0.701 | 0.680 | 0.660 | 0.716 | 0.702 | 0.547 | 0.651 |
|  | Headache | 0.814 | 0.809 | 0.905 | 0.996 | 0.702 | 0.692 | 0.652 | 0.662 | 0.605 | 0.605 | 0.562 | 0.594 |
|  | Nausea | 0.875 | 0.809 | 0.874 | 0.978 | 0.724 | 0.670 | 0.675 | 0.655 | 0.616 | 0.636 | 0.553 | 0.615 |
|  | Chills | 0.804 | 0.794 | 0.594 | 0.995 | 1.000 | 0.563 | 0.486 | 0.482 | 0.505 | 0.520 | 0.549 | 0.506 |
|  | Joint Pain | 0.868 | 0.843 | 0.797 | 0.997 | 1.000 | 0.728 | 0.672 | 0.674 | 0.643 | 0.660 | 0.631 | 0.635 |
|  | Muscle Pain | 0.864 | 0.821 | 0.988 | 0.978 | 0.718 | 0.735 | 0.630 | 0.637 | 0.638 | 0.648 | 0.594 | 0.612 |
|  | Local side effects | 0.871 | 0.828 | 0.993 | 0.994 | 0.786 | 0.649 | 0.664 | 0.535 | 0.707 | 0.725 | 0.693 | 0.622 |
| mRNA-1273 - Dose 2 | Fever | 0.809 | 0.819 | 0.883 | 0.975 | 1.000 | 0.722 | 0.683 | 0.622 | 0.689 | 0.709 | 0.571 | 0.689 |
|  | Fatigue | 0.792 | 0.796 | 0.827 | 0.954 | 1.000 | 0.752 | 0.717 | 0.711 | 0.763 | 0.762 | 0.657 | 0.701 |
|  | Headache | 0.830 | 0.796 | 0.978 | 0.997 | 0.774 | 0.758 | 0.744 | 0.737 | 0.735 | 0.785 | 0.706 | 0.748 |
|  | Nausea | 0.913 | 0.904 | 0.905 | 0.997 | 0.840 | 0.763 | 0.715 | 0.649 | 0.787 | 0.760 | 0.643 | 0.725 |
|  | Chills | 0.806 | 0.827 | 0.857 | 0.999 | 1.000 | 0.702 | 0.721 | 0.662 | 0.648 | 0.698 | 0.615 | 0.690 |
|  | Joint Pain | 0.814 | 0.855 | 0.979 | 0.979 | 1.000 | 0.791 | 0.734 | 0.684 | 0.785 | 0.774 | 0.708 | 0.731 |
|  | Muscle Pain | 0.865 | 0.849 | 0.856 | 0.986 | 0.782 | 0.754 | 0.776 | 0.742 | 0.759 | 0.771 | 0.709 | 0.717 |
|  | Local side effects | 0.900 | 0.879 | 1.000 | 0.982 | 0.842 | 0.835 | 0.850 | 0.815 | 0.771 | 0.824 | 0.773 | 0.810 |

**Supp Table 6. Full performance of Dose 2 with 46 parameters:**

The AUC performance of Second dose predictions without the inclusion of first dose side effects as inputs.

|  |  | AZD1222 | | | | | | | | Sputnik V | | | | | | | | BBIBP-CorV | | | | | | | |
| --- | --- | --- | --- | --- | --- | --- | --- | --- | --- | --- | --- | --- | --- | --- | --- | --- | --- | --- | --- | --- | --- | --- | --- | --- | --- |
|  |  | Fever | Fatigue | Headache | Nausea | Chills | Joint Pain | Muscle Pain | Local side effects | Fever | Fatigue | Headache | Nausea | Chills | Joint Pain | Muscle Pain | Local side effects | Fever | Fatigue | Headache | Nausea | Chills | Joint Pain | Muscle Pain | Local side effects |
| Training Set | Logistic Regression | 0.682 | 0.678 | 0.686 | 0.731 | 0.654 | 0.639 | 0.660 | 0.675 | 0.691 | 0.685 | 0.725 | 0.710 | 0.723 | 0.699 | 0.711 | 0.755 | 0.657 | 0.660 | 0.676 | 0.704 | 0.670 | 0.675 | 0.660 | 0.677 |
|  | SVM | 0.642 | 0.673 | 0.664 | 0.596 | 0.579 | 0.611 | 0.637 | 0.668 | 0.536 | 0.641 | 0.639 | 0.593 | 0.586 | 0.607 | 0.570 | 0.739 | 0.580 | 0.659 | 0.638 | 0.562 | 0.564 | 0.616 | 0.642 | 0.673 |
|  | XGBClassifier | 0.975 | 0.935 | 0.790 | 0.833 | 0.752 | 0.970 | 0.780 | 0.838 | 0.802 | 0.970 | 0.880 | 0.890 | 0.798 | 0.840 | 0.927 | 0.847 | 0.840 | 0.878 | 0.821 | 0.960 | 0.923 | 0.908 | 0.815 | 0.752 |
|  | RF | 0.859 | 0.897 | 0.760 | 0.907 | 0.909 | 0.825 | 0.853 | 0.871 | 0.874 | 0.824 | 0.922 | 0.991 | 0.893 | 0.884 | 0.893 | 0.885 | 0.854 | 0.845 | 0.804 | 0.923 | 0.858 | 0.844 | 0.837 | 0.849 |
|  | KNN | 1.000 | 1.000 | 1.000 | 1.000 | 1.000 | 1.000 | 1.000 | 1.000 | 1.000 | 1.000 | 1.000 | 1.000 | 1.000 | 1.000 | 1.000 | 1.000 | 1.000 | 1.000 | 1.000 | 1.000 | 1.000 | 1.000 | 1.000 | 1.000 |
|  | MLP | 0.661 | 0.658 | 0.678 | 0.716 | 0.606 | 0.626 | 0.640 | 0.671 | 0.659 | 0.677 | 0.705 | 0.655 | 0.672 | 0.682 | 0.695 | 0.747 | 0.621 | 0.652 | 0.667 | 0.667 | 0.649 | 0.661 | 0.650 | 0.673 |
| Validation Set | Logistic Regression | 0.641 | 0.637 | 0.651 | 0.693 | 0.605 | 0.599 | 0.618 | 0.645 | 0.643 | 0.657 | 0.695 | 0.643 | 0.672 | 0.650 | 0.674 | 0.731 | 0.630 | 0.639 | 0.658 | 0.666 | 0.634 | 0.646 | 0.637 | 0.664 |
|  | SVM | 0.613 | 0.628 | 0.622 | 0.591 | 0.554 | 0.574 | 0.598 | 0.638 | 0.513 | 0.630 | 0.600 | 0.510 | 0.560 | 0.574 | 0.539 | 0.712 | 0.563 | 0.637 | 0.628 | 0.554 | 0.545 | 0.597 | 0.616 | 0.661 |
|  | XGBClassifier | 0.618 | 0.630 | 0.634 | 0.702 | 0.613 | 0.590 | 0.610 | 0.642 | 0.643 | 0.645 | 0.677 | 0.653 | 0.668 | 0.652 | 0.659 | 0.718 | 0.643 | 0.631 | 0.650 | 0.663 | 0.649 | 0.646 | 0.648 | 0.655 |
|  | RF | 0.648 | 0.653 | 0.659 | 0.718 | 0.635 | 0.611 | 0.620 | 0.656 | 0.654 | 0.663 | 0.709 | 0.662 | 0.674 | 0.671 | 0.687 | 0.738 | 0.650 | 0.651 | 0.665 | 0.680 | 0.658 | 0.656 | 0.655 | 0.672 |
|  | KNN | 0.613 | 0.645 | 0.635 | 0.692 | 0.600 | 0.600 | 0.607 | 0.629 | 0.570 | 0.611 | 0.665 | 0.557 | 0.610 | 0.610 | 0.635 | 0.691 | 0.610 | 0.632 | 0.627 | 0.619 | 0.618 | 0.634 | 0.623 | 0.652 |
|  | MLP | 0.628 | 0.635 | 0.652 | 0.691 | 0.591 | 0.600 | 0.607 | 0.641 | 0.630 | 0.653 | 0.684 | 0.626 | 0.645 | 0.649 | 0.662 | 0.727 | 0.611 | 0.633 | 0.653 | 0.648 | 0.615 | 0.631 | 0.628 | 0.660 |
| Test Set | Logistic Regression | 0.636 | 0.611 | 0.670 | 0.664 | 0.614 | 0.610 | 0.647 | 0.629 | 0.622 | 0.623 | 0.646 | 0.630 | 0.555 | 0.677 | 0.708 | 0.747 | 0.654 | 0.634 | 0.668 | 0.661 | 0.640 | 0.641 | 0.659 | 0.639 |
|  | SVM | 0.592 | 0.620 | 0.666 | 0.573 | 0.551 | 0.596 | 0.619 | 0.638 | 0.549 | 0.598 | 0.577 | 0.484 | 0.499 | 0.514 | 0.536 | 0.722 | 0.448 | 0.636 | 0.642 | 0.564 | 0.451 | 0.580 | 0.645 | 0.635 |
|  | XGBClassifier | 0.614 | 0.605 | 0.653 | 0.688 | 0.629 | 0.596 | 0.607 | 0.613 | 0.593 | 0.595 | 0.661 | 0.620 | 0.596 | 0.683 | 0.697 | 0.735 | 0.641 | 0.636 | 0.671 | 0.671 | 0.653 | 0.658 | 0.650 | 0.635 |
|  | RF | 0.654 | 0.616 | 0.666 | 0.709 | 0.671 | 0.599 | 0.636 | 0.648 | 0.608 | 0.610 | 0.680 | 0.614 | 0.621 | 0.684 | 0.706 | 0.753 | 0.670 | 0.661 | 0.681 | 0.673 | 0.663 | 0.651 | 0.673 | 0.656 |
|  | KNN | 0.668 | 0.606 | 0.657 | 0.675 | 0.651 | 0.608 | 0.632 | 0.647 | 0.596 | 0.588 | 0.668 | 0.554 | 0.528 | 0.630 | 0.685 | 0.710 | 0.608 | 0.632 | 0.665 | 0.621 | 0.631 | 0.644 | 0.640 | 0.652 |
|  | MLP | 0.638 | 0.591 | 0.674 | 0.659 | 0.621 | 0.605 | 0.625 | 0.600 | 0.622 | 0.621 | 0.651 | 0.572 | 0.532 | 0.685 | 0.692 | 0.746 | 0.637 | 0.632 | 0.671 | 0.665 | 0.641 | 0.638 | 0.660 | 0.634 |

**Supp Table 7. Feature Importance:**

Detailed presentation of each input’s predictive value on all eight side effects for the AZD1222, Sputnik V and BBIBP-CorV vaccines

| **AstraZeneca D1** | **Dose1_Fever** | **Dose1_Fatigue** | **Dose1_Headache** | **Dose1_Nausea** | **Dose1_Chills** | **Dose1_Joint_Pain** | **Dose1_Muscle_Pain** | **Dose1_ Local side effects** | **Avg** |
| --- | --- | --- | --- | --- | --- | --- | --- | --- | --- |
| **Sex** | -0.242 | -0.310 | -0.527 | -1.056 | -0.275 | -0.250 | -0.317 | -0.796 | 0.472 |
| **Age** | -3.380 | -2.618 | -1.503 | -1.187 | -2.869 | -2.162 | -1.935 | -2.582 | 2.279 |
| **A** | 0.026 | -0.059 | 0.143 | -0.192 | -0.030 | -0.002 | -0.087 | 0.014 | 0.069 |
| **AB** | 0.051 | 0.023 | 0.206 | -0.013 | 0.039 | 0.183 | 0.165 | 0.039 | 0.090 |
| **B** | -0.029 | 0.082 | 0.018 | -0.240 | 0.000 | -0.064 | 0.002 | -0.031 | 0.058 |
| **O** | -0.047 | -0.044 | 0.027 | -0.261 | -0.007 | -0.020 | -0.080 | -0.022 | 0.064 |
| **Smoking** | 0.201 | 0.304 | -0.058 | 0.202 | 0.134 | 0.155 | 0.281 | -0.029 | 0.170 |
| **Substance use** | -0.320 | -0.001 | -0.073 | -0.115 | -0.293 | 0.048 | 0.040 | -0.239 | 0.141 |
| **Alcohol dependancy** | -0.134 | 0.015 | -0.056 | 0.146 | 0.141 | 0.420 | 0.200 | 0.424 | 0.192 |
| **Background Diabetes** | 0.300 | 0.097 | 0.073 | 0.221 | -0.162 | -0.018 | 0.046 | 0.060 | 0.122 |
| **Background Cardiovascular disease** | -0.131 | -0.062 | 0.008 | 0.069 | -0.065 | 0.052 | -0.054 | -0.214 | 0.082 |
| **Background Hypertension** | 0.304 | 0.239 | -0.011 | -0.208 | 0.184 | 0.146 | 0.054 | 0.264 | 0.176 |
| **Background CancerPassive** | 0.427 | 0.457 | 0.185 | 0.163 | 0.024 | 0.214 | 0.324 | 0.302 | 0.262 |
| **Background CancerActive** | -0.393 | 0.225 | -0.019 | 0.081 | 0.037 | -0.369 | -0.032 | 0.533 | 0.211 |
| **Background Neurological** | 0.218 | 0.194 | 0.052 | 0.262 | 0.113 | 0.226 | 0.195 | -0.116 | 0.172 |
| **Background pulmonary** | 0.144 | 0.275 | 0.257 | -0.121 | 0.245 | 0.204 | 0.212 | 0.286 | 0.218 |
| **Background ImmuneSystem** | 0.063 | 0.277 | -0.020 | -0.150 | -0.143 | 0.411 | 0.266 | 0.140 | 0.184 |
| **Background hematologic** | -0.141 | 0.054 | 0.387 | 0.047 | 0.623 | 0.162 | -0.068 | 0.237 | 0.215 |
| **Background Gastrointestinal** | 0.140 | 0.435 | 0.301 | 0.276 | 0.410 | 0.321 | 0.144 | -0.164 | 0.274 |
| **Background Renal** | 0.246 | -0.038 | -0.043 | -0.162 | 0.493 | 0.215 | -0.002 | 0.285 | 0.185 |
| **Background Hepatic** | -0.194 | -0.217 | -0.042 | -0.250 | 0.233 | 0.269 | 0.039 | 0.338 | 0.198 |
| **Background Skeletal** | 0.113 | 0.057 | 0.155 | -0.149 | 0.120 | 0.408 | 0.173 | 0.085 | 0.157 |
| **Background Mental** | -0.073 | -0.460 | 0.157 | -0.043 | 0.161 | -0.195 | -0.073 | -0.221 | 0.173 |
| **Background Allergy** | 0.196 | 0.375 | 0.236 | 0.152 | 0.438 | 0.435 | 0.291 | 0.277 | 0.300 |
| **Background None** | 0.016 | 0.066 | 0.089 | 0.012 | 0.300 | 0.242 | 0.124 | 0.148 | 0.125 |
| **Hormonal med** | 0.077 | 0.193 | -0.021 | -0.121 | 0.043 | 0.053 | 0.157 | 0.474 | 0.142 |
| **Pregnancy** | 0.053 | 0.106 | 0.073 | -0.070 | 0.052 | -0.041 | -0.029 | 0.024 | 0.056 |
| **Respiratory Inhaler** | 0.009 | -0.076 | -0.025 | -0.085 | -0.199 | -0.151 | -0.005 | -0.138 | 0.086 |
| **Corticosteroid Med** | -0.076 | 0.178 | -0.153 | 0.262 | 0.369 | 0.072 | 0.030 | 0.341 | 0.185 |
| **Chemotherapy Med** | -0.018 | -0.237 | 0.063 | -0.003 | -0.201 | -0.177 | -0.243 | -0.162 | 0.138 |
| **Immunosuppressive Med** | -0.307 | -0.189 | -0.133 | -0.366 | -0.268 | -0.018 | -0.235 | -0.216 | 0.216 |
| **Covid Infection** | -0.588 | -1.017 | -0.704 | -0.461 | -0.518 | -0.572 | -0.567 | 0.016 | 0.555 |
| **Covid Fever** | 0.965 | 0.102 | -0.002 | 0.141 | 0.828 | -0.041 | 0.049 | -0.058 | 0.273 |
| **Covid Fatigue** | 0.017 | 0.836 | 0.001 | -0.084 | -0.067 | 0.446 | 0.527 | 0.081 | 0.258 |
| **Covid Cough** | 0.209 | -0.076 | 0.089 | 0.053 | -0.036 | 0.150 | 0.031 | 0.054 | 0.087 |
| **Covid Gastrointestinal** | 0.226 | 0.332 | 0.099 | 0.417 | 0.113 | 0.247 | 0.268 | 0.418 | 0.265 |
| **Covid Anosmia** | 0.040 | -0.051 | -0.096 | -0.010 | 0.125 | 0.089 | -0.021 | -0.001 | 0.054 |
| **Covid RespiratoryProblems** | -0.012 | 0.362 | 0.095 | -0.029 | -0.023 | 0.291 | 0.241 | 0.219 | 0.159 |
| **Covid ConsciousnessDisorder** | 0.372 | -0.071 | 0.190 | 0.488 | 0.258 | -0.039 | -0.174 | -0.056 | 0.206 |
| **Covid Paresis** | -0.349 | 0.145 | -0.064 | 0.050 | -0.228 | 0.384 | 0.092 | -0.229 | 0.193 |
| **Covid ChestPain** | 0.104 | -0.145 | 0.135 | 0.172 | 0.005 | 0.174 | 0.080 | 0.074 | 0.111 |
| **Covid Headache** | -0.043 | 0.186 | 0.933 | 0.181 | -0.085 | -0.019 | 0.138 | 0.060 | 0.206 |
| **Covid SoreThroat** | -0.279 | 0.160 | 0.133 | 0.009 | 0.088 | 0.068 | -0.058 | -0.026 | 0.103 |
| **Covid Vertigo** | 0.278 | 0.117 | 0.439 | 0.502 | 0.379 | 0.337 | 0.307 | -0.145 | 0.313 |
| **Covid hospitalization** | 0.014 | -0.059 | -0.097 | 0.060 | -0.177 | -0.169 | -0.235 | 0.021 | 0.104 |
| **BMI** | -1.717 | -0.767 | -0.141 | -0.304 | -0.961 | -0.139 | -0.151 | -0.871 | 0.632 |

| **AstraZeneca D2** | **Dose2_Fever** | **Dose2_Fatigue** | **Dose2_Headache** | **Dose2_Nausea** | **Dose2_Chills** | **Dose2_Joint_Pain** | **Dose2_Muscle_Pain** | **Dose2_ Local side effects** | **Avg** |
| --- | --- | --- | --- | --- | --- | --- | --- | --- | --- |
| **Sex** | -0.071 | -0.286 | -0.351 | -0.532 | -0.128 | -0.138 | -0.188 | -0.319 | 0.252 |
| **Age** | -1.143 | -0.093 | -0.151 | -0.251 | -0.194 | 0.069 | -0.467 | -0.186 | 0.319 |
| **A** | -0.102 | -0.186 | -0.071 | -0.054 | -0.071 | -0.032 | -0.388 | -0.039 | 0.118 |
| **AB** | 0.233 | 0.098 | 0.125 | 0.164 | 0.080 | 0.136 | -0.228 | 0.005 | 0.134 |
| **B** | -0.040 | -0.044 | -0.093 | -0.098 | -0.030 | -0.170 | -0.375 | -0.068 | 0.115 |
| **O** | -0.078 | 0.131 | 0.040 | -0.012 | 0.019 | 0.066 | -0.305 | -0.050 | 0.088 |
| **Smoking** | 0.065 | 0.280 | 0.116 | -0.130 | 0.044 | 0.124 | 0.185 | -0.017 | 0.120 |
| **Substance use** | -0.822 | 0.076 | 0.021 | -0.076 | 0.008 | 0.017 | 0.126 | -0.010 | 0.145 |
| **Alcohol dependancy** | -0.523 | 0.130 | -0.098 | 0.001 | 0.051 | 0.091 | -0.030 | -0.002 | 0.116 |
| **Background Diabetes** | -0.234 | 0.115 | -0.155 | -0.167 | -0.234 | 0.089 | -0.052 | -0.027 | 0.134 |
| **Background Cardiovascular disease** | -0.107 | 0.091 | 0.139 | -0.258 | -0.007 | 0.237 | 0.215 | -0.044 | 0.137 |
| **Background Hypertension** | 0.056 | -0.092 | -0.169 | -0.025 | 0.081 | -0.029 | -0.249 | -0.113 | 0.102 |
| **Background CancerPassive** | 0.410 | 0.136 | 0.127 | 0.072 | 0.042 | 0.042 | 0.020 | 0.016 | 0.108 |
| **Background CancerActive** | -0.406 | -0.172 | -0.148 | -0.028 | -0.038 | -0.068 | -0.148 | -0.019 | 0.128 |
| **Background Neurological** | 0.761 | -0.094 | 0.286 | 0.298 | 0.174 | -0.007 | 0.073 | -0.004 | 0.212 |
| **Background pulmonary** | 0.686 | 0.060 | 0.147 | 0.009 | 0.266 | 0.023 | 0.047 | -0.028 | 0.158 |
| **Background ImmuneSystem** | 0.295 | -0.103 | -0.162 | -0.092 | 0.119 | 0.277 | 0.057 | 0.019 | 0.140 |
| **Background hematologic** | 0.627 | 0.241 | 0.019 | -0.024 | 0.274 | 0.085 | -0.090 | 0.026 | 0.173 |
| **Background Gastrointestinal** | -0.219 | 0.134 | -0.085 | 0.147 | -0.023 | 0.240 | -0.094 | -0.001 | 0.118 |
| **Background Renal** | 0.469 | -0.022 | 0.043 | -0.052 | -0.082 | -0.086 | -0.029 | -0.028 | 0.101 |
| **Background Hepatic** | 0.205 | -0.259 | -0.105 | 0.044 | 0.258 | 0.148 | -0.061 | -0.006 | 0.136 |
| **Background Skeletal** | -0.264 | 0.025 | 0.001 | 0.014 | -0.091 | 0.133 | 0.117 | 0.015 | 0.082 |
| **Background Mental** | -0.380 | -0.214 | -0.079 | 0.139 | -0.109 | -0.111 | -0.098 | 0.001 | 0.142 |
| **Background Allergy** | 0.329 | 0.115 | 0.059 | 0.058 | -0.019 | 0.034 | -0.258 | 0.040 | 0.114 |
| **Background None** | 0.207 | -0.029 | 0.051 | -0.040 | 0.156 | 0.175 | -0.189 | -0.087 | 0.117 |
| **Hormonal med** | -0.050 | -0.063 | -0.099 | 0.140 | -0.076 | 0.030 | -0.003 | 0.060 | 0.065 |
| **Pregnancy** | 0.427 | 0.176 | 0.119 | -0.002 | -0.030 | 0.271 | 0.292 | 0.040 | 0.170 |
| **Respiratory Inhaler** | -0.028 | -0.099 | -0.096 | 0.046 | 0.051 | -0.016 | -0.153 | -0.101 | 0.074 |
| **Corticosteroid Med** | -0.473 | -0.311 | -0.294 | -0.167 | -0.197 | -0.146 | -0.225 | -0.053 | 0.233 |
| **Chemotherapy Med** | 0.461 | 0.188 | 0.121 | 0.097 | -0.021 | 0.114 | 0.105 | -0.093 | 0.150 |
| **Immunosuppressive Med** | -0.181 | -0.180 | -0.074 | -0.042 | 0.072 | -0.141 | -0.201 | -0.092 | 0.123 |
| **Covid Infection** | -1.223 | -0.239 | -0.302 | -0.265 | -0.310 | -0.200 | -0.326 | 0.027 | 0.361 |
| **Covid Fever** | 0.602 | -0.051 | 0.087 | 0.280 | 0.343 | 0.085 | -0.162 | -0.013 | 0.203 |
| **Covid Fatigue** | 0.671 | 0.347 | 0.133 | 0.000 | 0.185 | 0.204 | 0.290 | 0.071 | 0.238 |
| **Covid Cough** | -0.130 | -0.077 | -0.008 | 0.306 | -0.021 | 0.069 | 0.102 | 0.048 | 0.095 |
| **Covid Gastrointestinal** | 0.006 | 0.215 | -0.109 | 0.433 | -0.039 | 0.111 | 0.104 | 0.044 | 0.133 |
| **Covid Anosmia** | -0.086 | -0.425 | -0.391 | 0.039 | -0.147 | -0.060 | -0.107 | 0.050 | 0.163 |
| **Covid RespiratoryProblems** | 0.030 | 0.129 | 0.308 | -0.070 | 0.141 | 0.109 | 0.082 | 0.031 | 0.112 |
| **Covid ConsciousnessDisorder** | -0.204 | -0.061 | 0.164 | 0.257 | -0.011 | 0.050 | -0.013 | 0.006 | 0.096 |
| **Covid Paresis** | 0.242 | 0.224 | 0.038 | 0.128 | 0.142 | 0.034 | 0.279 | -0.010 | 0.137 |
| **Covid ChestPain** | 0.275 | -0.004 | 0.298 | 0.160 | 0.178 | 0.088 | 0.303 | 0.007 | 0.164 |
| **Covid Headache** | 0.181 | 0.170 | 0.588 | 0.054 | 0.187 | -0.134 | 0.254 | 0.073 | 0.205 |
| **Covid SoreThroat** | 0.496 | 0.368 | 0.225 | 0.186 | 0.222 | 0.312 | 0.188 | 0.046 | 0.255 |
| **Covid Vertigo** | 0.018 | 0.286 | 0.206 | 0.001 | -0.008 | 0.160 | 0.152 | -0.043 | 0.109 |
| **Covid hospitalization** | -0.040 | -0.137 | 0.191 | 0.230 | -0.003 | -0.004 | -0.079 | -0.020 | 0.088 |
| **Dose1_Fever** | 2.401 | -0.011 | 0.082 | 0.084 | 0.099 | -0.217 | -0.180 | 0.088 | 0.395 |
| **Dose1_Fatigue** | 0.133 | 2.071 | 0.424 | 0.286 | 0.229 | 0.282 | 0.400 | 0.122 | 0.493 |
| **Dose1_Headache** | 0.185 | 0.271 | 2.263 | 0.331 | 0.083 | 0.101 | 0.191 | 0.011 | 0.429 |
| **Dose1_Nausea** | -0.094 | 0.165 | 0.205 | 2.391 | -0.037 | 0.011 | -0.044 | 0.011 | 0.370 |
| **Dose1_Chills** | -0.091 | 0.002 | -0.123 | 0.269 | 1.923 | 0.098 | -0.158 | -0.022 | 0.336 |
| **Dose1_Joint_Pain** | 0.053 | 0.120 | 0.281 | 0.031 | 0.196 | 2.162 | 0.644 | 0.077 | 0.446 |
| **Dose1_Muscle_Pain** | -0.039 | 0.220 | -0.198 | -0.003 | -0.089 | 0.515 | 1.757 | 0.128 | 0.369 |
| **Dose1_ Local side effects** | 0.062 | 0.133 | 0.057 | -0.042 | 0.012 | 0.157 | 0.059 | 1.169 | 0.211 |
| **BMI** | 0.371 | 0.130 | 0.258 | 0.186 | 0.116 | 0.063 | -0.134 | -0.051 | 0.164 |

| **SputnikV D1** | **Dose1_Fever** | **Dose1_Fatigue** | **Dose1_Headache** | **Dose1_Nausea** | **Dose1_Chills** | **Dose1_Joint_Pain** | **Dose1_Muscle_Pain** | **Dose1_ Local side effects** | **Avg** |
| --- | --- | --- | --- | --- | --- | --- | --- | --- | --- |
| **Sex** | -0.251 | -0.411 | -0.363 | -0.964 | -0.207 | -0.211 | -0.203 | -0.890 | 0.438 |
| **Age** | -4.277 | -2.660 | -2.614 | -2.026 | -4.895 | -4.156 | -3.651 | -2.087 | 3.296 |
| **A** | 0.126 | 0.262 | 0.014 | -0.033 | 0.126 | -0.019 | 0.019 | 0.068 | 0.083 |
| **AB** | -0.147 | 0.053 | 0.079 | 0.082 | 0.018 | 0.073 | -0.047 | -0.111 | 0.076 |
| **B** | -0.018 | 0.132 | 0.018 | -0.134 | -0.082 | -0.058 | -0.017 | 0.007 | 0.058 |
| **O** | 0.044 | 0.234 | 0.106 | 0.085 | 0.131 | 0.007 | 0.040 | 0.037 | 0.085 |
| **Smoking** | 0.014 | 0.072 | -0.199 | 0.158 | 0.148 | 0.123 | -0.019 | -0.019 | 0.094 |
| **Substance use** | 0.248 | 0.461 | -0.239 | -0.356 | -0.822 | 0.228 | 0.719 | -0.084 | 0.395 |
| **Alcohol dependancy** | -0.085 | -0.118 | 0.079 | 0.291 | 0.441 | 0.034 | 0.046 | 0.353 | 0.181 |
| **Background Diabetes** | -0.074 | -0.269 | -0.057 | -0.061 | -0.145 | 0.011 | -0.248 | -0.041 | 0.113 |
| **Background Cardiovascular disease** | 0.098 | -0.092 | -0.023 | -0.244 | 0.009 | 0.005 | 0.054 | -0.042 | 0.071 |
| **Background Hypertension** | 0.147 | 0.223 | -0.096 | 0.087 | -0.104 | 0.197 | 0.164 | 0.204 | 0.153 |
| **Background CancerPassive** | 0.263 | -0.166 | -0.198 | 0.306 | 0.424 | 0.512 | -0.207 | -0.147 | 0.278 |
| **Background CancerActive** | -0.132 | 0.057 | 0.256 | 0.216 | 0.947 | 0.602 | 0.400 | -0.111 | 0.340 |
| **Background Neurological** | 0.063 | 0.579 | -0.097 | 0.490 | 0.309 | 0.247 | 0.270 | -0.076 | 0.266 |
| **Background pulmonary** | 0.372 | -0.096 | 0.058 | 0.374 | -0.034 | 0.023 | 0.075 | -0.145 | 0.147 |
| **Background ImmuneSystem** | 0.066 | 0.173 | -0.221 | -0.040 | 0.323 | 0.173 | 0.178 | 0.117 | 0.161 |
| **Background hematologic** | 0.384 | -0.116 | -0.033 | -0.004 | 0.048 | 0.304 | -0.183 | -0.109 | 0.148 |
| **Background Gastrointestinal** | 0.339 | 0.407 | 0.395 | 0.461 | 0.463 | 0.346 | 0.411 | 0.183 | 0.376 |
| **Background Renal** | 0.596 | 0.024 | 0.091 | 0.382 | 0.386 | 0.591 | 0.289 | 0.158 | 0.315 |
| **Background Hepatic** | 0.209 | -0.045 | -0.139 | 0.174 | -0.278 | -0.149 | -0.227 | -0.313 | 0.192 |
| **Background Skeletal** | 0.249 | 0.200 | 0.114 | 0.161 | 0.241 | 0.516 | 0.283 | 0.167 | 0.241 |
| **Background Mental** | -0.113 | -0.038 | -0.215 | -0.541 | -0.240 | 0.134 | -0.046 | -0.220 | 0.193 |
| **Background Allergy** | 0.266 | 0.263 | 0.056 | 0.146 | 0.292 | 0.149 | 0.206 | 0.244 | 0.203 |
| **Background None** | 0.145 | 0.065 | -0.101 | 0.021 | 0.096 | 0.069 | 0.027 | 0.056 | 0.073 |
| **Hormonal med** | 0.049 | 0.396 | 0.568 | 0.446 | 0.192 | 0.052 | 0.371 | 0.218 | 0.287 |
| **Pregnancy** | 0.028 | -0.023 | -0.043 | -0.248 | 0.175 | 0.069 | 0.238 | -0.122 | 0.118 |
| **Respiratory Inhaler** | -0.088 | 0.119 | 0.231 | 0.403 | -0.036 | 0.169 | -0.094 | 0.052 | 0.149 |
| **Corticosteroid Med** | 0.025 | 0.053 | -0.095 | 0.119 | 0.020 | -0.086 | 0.002 | 0.023 | 0.053 |
| **Chemotherapy Med** | -0.223 | -0.259 | -0.129 | -1.309 | -0.201 | -0.100 | -0.060 | -0.274 | 0.319 |
| **Immunosuppressive Med** | -0.082 | -0.079 | -0.272 | 0.316 | 0.031 | 0.007 | -0.079 | -0.305 | 0.146 |
| **Covid Infection** | -0.591 | -0.839 | -0.875 | -0.812 | -0.683 | -0.954 | -0.884 | -0.263 | 0.738 |
| **Covid Fever** | 0.985 | 0.197 | 0.167 | 0.033 | 0.907 | 0.349 | 0.219 | 0.189 | 0.381 |
| **Covid Fatigue** | 0.198 | 0.950 | 0.240 | 0.099 | 0.295 | 0.755 | 1.008 | 0.299 | 0.481 |
| **Covid Cough** | -0.039 | -0.109 | 0.175 | -0.041 | 0.157 | -0.142 | 0.004 | -0.133 | 0.100 |
| **Covid Gastrointestinal** | 0.048 | 0.314 | 0.080 | 0.474 | -0.028 | 0.171 | 0.333 | 0.105 | 0.194 |
| **Covid Anosmia** | -0.036 | 0.088 | -0.102 | 0.081 | -0.008 | 0.107 | 0.126 | 0.113 | 0.083 |
| **Covid RespiratoryProblems** | 0.096 | 0.064 | -0.050 | 0.003 | -0.092 | 0.091 | -0.023 | 0.344 | 0.095 |
| **Covid ConsciousnessDisorder** | 0.510 | 0.216 | 0.604 | 0.355 | 0.147 | 0.760 | 0.075 | -0.132 | 0.350 |
| **Covid Paresis** | -0.058 | 0.294 | 0.524 | 0.286 | 0.168 | 0.811 | 0.412 | -0.150 | 0.338 |
| **Covid ChestPain** | -0.053 | 0.312 | 0.092 | 0.121 | -0.025 | 0.280 | 0.250 | 0.218 | 0.169 |
| **Covid Headache** | 0.172 | 0.328 | 1.193 | 0.593 | 0.184 | 0.317 | 0.263 | 0.368 | 0.427 |
| **Covid SoreThroat** | -0.147 | -0.317 | -0.163 | -0.212 | -0.335 | -0.196 | -0.221 | -0.148 | 0.217 |
| **Covid Vertigo** | 0.307 | 0.173 | 0.402 | 0.578 | 0.309 | 0.350 | 0.294 | -0.003 | 0.302 |
| **Covid hospitalization** | 0.207 | 0.004 | 0.003 | 0.368 | 0.000 | -0.147 | -0.273 | 0.119 | 0.140 |
| **BMI** | -0.542 | 0.127 | -0.080 | -0.135 | -2.124 | -0.298 | -0.111 | 0.007 | 0.428 |

| **SputnikV D2** | **Dose2_Fever** | **Dose2_Fatigue** | **Dose2_Headache** | **Dose2_Nausea** | **Dose2_Chills** | **Dose2_Joint_Pain** | **Dose2_Muscle_Pain** | **Dose2_ Local side effects** | **Avg** |
| --- | --- | --- | --- | --- | --- | --- | --- | --- | --- |
| **Sex** | 0.071 | 0.053 | -0.180 | -0.541 | -0.150 | -0.151 | -0.043 | -0.412 | 0.200 |
| **Age** | -0.492 | -0.205 | -0.280 | -0.565 | -0.549 | -0.652 | -0.072 | -0.169 | 0.373 |
| **A** | -0.384 | -0.123 | -0.403 | -0.587 | -0.421 | -0.340 | -0.048 | -0.054 | 0.295 |
| **AB** | -0.328 | -0.063 | -0.260 | -0.243 | -0.349 | -0.289 | -0.001 | -0.032 | 0.196 |
| **B** | -0.477 | 0.045 | -0.382 | -0.497 | -0.307 | -0.365 | -0.039 | 0.043 | 0.269 |
| **O** | -0.374 | 0.142 | -0.324 | -0.399 | -0.282 | -0.350 | 0.088 | 0.044 | 0.250 |
| **Smoking** | 0.036 | 0.037 | 0.033 | -0.221 | 0.084 | -0.065 | -0.031 | 0.100 | 0.076 |
| **Substance use** | 0.314 | 0.072 | 0.066 | 0.023 | -0.092 | 0.055 | 0.011 | -0.077 | 0.089 |
| **Alcohol dependancy** | 0.746 | 0.069 | 0.028 | 0.114 | 0.243 | 0.116 | 0.031 | 0.021 | 0.171 |
| **Background Diabetes** | -0.041 | -0.115 | 0.032 | -0.366 | -0.111 | -0.208 | -0.064 | 0.135 | 0.134 |
| **Background Cardiovascular disease** | -0.298 | 0.045 | -0.121 | -0.047 | -0.143 | -0.276 | -0.004 | -0.331 | 0.158 |
| **Background Hypertension** | 0.181 | -0.058 | -0.078 | -0.273 | 0.059 | -0.083 | -0.048 | 0.101 | 0.110 |
| **Background CancerPassive** | -0.241 | 0.193 | 0.134 | -0.287 | -0.205 | -0.095 | -0.017 | 0.132 | 0.163 |
| **Background CancerActive** | -0.279 | -0.025 | 0.033 | -0.050 | 0.095 | -0.038 | 0.003 | -0.039 | 0.070 |
| **Background Neurological** | 0.102 | -0.072 | 0.017 | -0.282 | 0.068 | -0.065 | 0.045 | -0.087 | 0.092 |
| **Background pulmonary** | 0.249 | 0.172 | 0.242 | -0.003 | 0.090 | 0.227 | 0.056 | -0.273 | 0.164 |
| **Background ImmuneSystem** | -0.028 | 0.173 | 0.021 | 0.116 | 0.088 | -0.115 | 0.035 | 0.208 | 0.098 |
| **Background hematologic** | 0.747 | -0.077 | 0.105 | 0.077 | 0.268 | 0.035 | 0.025 | 0.224 | 0.195 |
| **Background Gastrointestinal** | -0.053 | 0.105 | -0.103 | -0.104 | 0.012 | 0.035 | 0.056 | 0.295 | 0.095 |
| **Background Renal** | 0.301 | 0.257 | 0.132 | 0.045 | 0.093 | 0.240 | 0.007 | 0.088 | 0.145 |
| **Background Hepatic** | 0.065 | -0.004 | -0.077 | -0.024 | 0.080 | -0.105 | 0.004 | -0.077 | 0.055 |
| **Background Skeletal** | -0.017 | -0.012 | -0.139 | 0.079 | -0.143 | 0.238 | 0.031 | -0.190 | 0.106 |
| **Background Mental** | 0.792 | 0.318 | 0.083 | 0.007 | 0.235 | 0.183 | 0.056 | 0.113 | 0.223 |
| **Background Allergy** | 0.226 | 0.010 | 0.043 | -0.092 | -0.178 | -0.042 | 0.061 | 0.133 | 0.098 |
| **Background None** | -0.006 | -0.120 | -0.194 | -0.352 | -0.079 | -0.251 | -0.052 | -0.111 | 0.146 |
| **Hormonal med** | 0.460 | 0.365 | -0.099 | -0.053 | 0.363 | 0.313 | 0.037 | 0.288 | 0.247 |
| **Pregnancy** | 0.431 | 0.379 | 0.115 | 0.220 | 0.244 | 0.326 | 0.113 | 0.118 | 0.243 |
| **Respiratory Inhaler** | -0.247 | -0.152 | -0.151 | -0.053 | -0.157 | -0.166 | -0.032 | 0.021 | 0.122 |
| **Corticosteroid Med** | 0.103 | 0.107 | -0.204 | -0.305 | -0.092 | -0.092 | -0.007 | -0.050 | 0.120 |
| **Chemotherapy Med** | 0.201 | -0.143 | 0.129 | 0.115 | 0.165 | 0.128 | -0.021 | -0.189 | 0.136 |
| **Immunosuppressive Med** | -0.286 | -0.042 | 0.111 | -0.047 | -0.042 | 0.020 | -0.029 | 0.027 | 0.076 |
| **Covid Infection** | -0.232 | -0.192 | -0.221 | -0.421 | -0.065 | -0.310 | -0.069 | -0.302 | 0.226 |
| **Covid Fever** | 0.237 | -0.131 | -0.071 | 0.130 | 0.180 | -0.074 | 0.019 | -0.111 | 0.119 |
| **Covid Fatigue** | -0.060 | 0.434 | 0.054 | -0.243 | 0.071 | 0.335 | 0.158 | 0.275 | 0.204 |
| **Covid Cough** | 0.012 | 0.003 | -0.127 | -0.101 | -0.014 | -0.077 | 0.024 | 0.042 | 0.050 |
| **Covid Gastrointestinal** | 0.281 | 0.063 | -0.190 | 0.341 | 0.076 | 0.076 | 0.026 | -0.036 | 0.136 |
| **Covid Anosmia** | 0.026 | 0.079 | -0.072 | 0.271 | -0.048 | -0.061 | -0.024 | 0.117 | 0.087 |
| **Covid RespiratoryProblems** | 0.090 | 0.136 | 0.092 | 0.141 | 0.210 | 0.139 | 0.023 | 0.112 | 0.118 |
| **Covid ConsciousnessDisorder** | -0.024 | 0.105 | -0.052 | -0.129 | 0.099 | 0.144 | -0.023 | -0.083 | 0.082 |
| **Covid Paresis** | -0.133 | -0.087 | -0.119 | 0.074 | 0.027 | 0.131 | 0.015 | -0.006 | 0.074 |
| **Covid ChestPain** | 0.109 | -0.112 | 0.125 | 0.292 | -0.123 | 0.366 | 0.047 | 0.140 | 0.164 |
| **Covid Headache** | 0.056 | 0.093 | 0.814 | 0.037 | 0.002 | 0.024 | -0.010 | 0.094 | 0.141 |
| **Covid SoreThroat** | -0.091 | 0.013 | -0.090 | -0.067 | -0.017 | -0.059 | 0.013 | 0.162 | 0.064 |
| **Covid Vertigo** | -0.087 | 0.240 | 0.305 | 0.286 | -0.077 | 0.118 | 0.062 | -0.123 | 0.162 |
| **Covid hospitalization** | 0.102 | -0.126 | -0.353 | 0.061 | 0.111 | -0.148 | -0.028 | -0.031 | 0.120 |
| **Dose1_Fever** | 2.776 | -0.044 | 0.191 | 0.121 | 0.105 | -0.281 | 0.145 | -0.004 | 0.458 |
| **Dose1_Fatigue** | 0.051 | 2.269 | 0.326 | -0.168 | 0.070 | 0.230 | 0.449 | 0.266 | 0.479 |
| **Dose1_Headache** | 0.122 | 0.435 | 2.587 | 0.368 | 0.098 | 0.221 | 0.298 | 0.153 | 0.535 |
| **Dose1_Nausea** | 0.272 | 0.237 | 0.247 | 2.928 | 0.011 | 0.217 | 0.079 | -0.211 | 0.525 |
| **Dose1_Chills** | -0.060 | -0.125 | -0.097 | 0.079 | 2.217 | -0.062 | 0.089 | -0.144 | 0.359 |
| **Dose1_Joint_Pain** | 0.054 | 0.122 | -0.013 | 0.181 | 0.181 | 2.582 | 0.489 | 0.023 | 0.456 |
| **Dose1_Muscle_Pain** | -0.159 | 0.439 | 0.126 | 0.064 | 0.005 | 0.483 | 1.445 | 0.069 | 0.349 |
| **Dose1_ Local side effects** | 0.105 | 0.032 | 0.012 | -0.158 | 0.074 | -0.150 | 0.099 | 2.750 | 0.422 |
| **BMI** | -0.925 | -0.364 | -0.259 | -0.345 | -0.449 | -0.150 | -0.008 | -0.143 | 0.330 |

| **Sinopharm D1** | **Dose1_Fever** | **Dose1_Fatigue** | **Dose1_Headache** | **Dose1_Nausea** | **Dose1_Chills** | **Dose1_Joint_Pain** | **Dose1_Muscle_Pain** | **Dose1_ Local side effects** | **Avg** |
| --- | --- | --- | --- | --- | --- | --- | --- | --- | --- |
| **Sex** | -0.631 | -0.725 | -0.710 | -0.963 | -0.826 | -0.760 | -0.663 | -0.860 | 0.767 |
| **Age** | -1.411 | -1.609 | -1.519 | -0.868 | -0.844 | -1.007 | -1.760 | -3.112 | 1.516 |
| **A** | -0.413 | -0.014 | -0.159 | -0.431 | -0.400 | -0.059 | -0.237 | 0.036 | 0.219 |
| **AB** | -0.139 | -0.063 | -0.022 | -0.305 | -0.231 | 0.059 | -0.103 | -0.044 | 0.121 |
| **B** | -0.285 | -0.007 | -0.192 | -0.327 | -0.539 | -0.054 | -0.174 | -0.002 | 0.197 |
| **O** | -0.179 | 0.063 | -0.083 | -0.510 | -0.410 | 0.056 | -0.104 | 0.010 | 0.177 |
| **Smoking** | -0.109 | 0.199 | -0.096 | -0.033 | -0.179 | 0.012 | -0.072 | -0.112 | 0.102 |
| **Substance use** | -0.055 | -0.083 | -0.033 | -0.137 | -0.054 | -0.326 | 0.029 | -0.681 | 0.175 |
| **Alcohol dependancy** | -0.056 | -0.240 | -0.236 | -0.017 | -0.070 | -0.694 | -0.816 | 0.173 | 0.288 |
| **Background Diabetes** | 0.040 | -0.131 | -0.364 | -0.167 | 0.007 | 0.145 | 0.032 | 0.018 | 0.113 |
| **Background Cardiovascular disease** | 0.324 | 0.357 | 0.177 | -0.204 | 0.083 | 0.147 | 0.159 | 0.241 | 0.212 |
| **Background Hypertension** | -0.101 | 0.154 | 0.065 | 0.041 | 0.015 | 0.015 | 0.306 | 0.007 | 0.088 |
| **Background CancerPassive** | 0.284 | 0.469 | 0.266 | -0.058 | -0.054 | 0.047 | 0.132 | 0.157 | 0.183 |
| **Background CancerActive** | -0.248 | -0.018 | 0.136 | 0.001 | 0.194 | 0.654 | 0.813 | -0.122 | 0.273 |
| **Background Neurological** | 0.621 | 0.123 | 0.192 | 0.117 | 0.331 | 0.597 | 0.390 | 0.103 | 0.309 |
| **Background pulmonary** | 0.216 | 0.356 | 0.171 | -0.036 | 0.272 | 0.805 | 0.558 | 0.039 | 0.307 |
| **Background ImmuneSystem** | 0.199 | 0.187 | -0.024 | -0.414 | -0.188 | 0.015 | -0.118 | 0.097 | 0.155 |
| **Background hematologic** | 0.252 | -0.204 | -0.138 | -0.152 | -0.119 | -0.197 | -0.210 | -0.253 | 0.191 |
| **Background Gastrointestinal** | 0.475 | 0.312 | 0.559 | -0.075 | 0.254 | 0.152 | 0.332 | 0.354 | 0.314 |
| **Background Renal** | 0.300 | 0.343 | -0.107 | 0.168 | 0.059 | 0.217 | -0.059 | 0.202 | 0.182 |
| **Background Hepatic** | 0.009 | 0.157 | 0.247 | -0.082 | 0.087 | 0.251 | 0.322 | -0.129 | 0.160 |
| **Background Skeletal** | -0.189 | -0.018 | 0.068 | -0.082 | -0.040 | 0.136 | -0.065 | 0.310 | 0.113 |
| **Background Mental** | 0.053 | -0.079 | -0.173 | -0.057 | 0.133 | 0.155 | 0.313 | -0.429 | 0.174 |
| **Background Allergy** | 0.245 | 0.445 | 0.142 | -0.116 | 0.241 | 0.151 | 0.153 | 0.162 | 0.207 |
| **Background None** | -0.172 | 0.032 | -0.057 | -0.389 | -0.299 | -0.225 | -0.228 | 0.109 | 0.189 |
| **Hormonal med** | -0.154 | 0.356 | 0.060 | 0.021 | 0.013 | 0.235 | -0.190 | 0.037 | 0.133 |
| **Pregnancy** | -0.345 | -0.056 | -0.047 | -0.254 | -0.229 | -0.092 | -0.083 | 0.095 | 0.150 |
| **Respiratory Inhaler** | -0.135 | -0.032 | 0.084 | 0.157 | 0.011 | -0.166 | -0.044 | 0.116 | 0.093 |
| **Corticosteroid Med** | 0.031 | -0.019 | 0.201 | -0.135 | 0.027 | -0.142 | 0.046 | 0.081 | 0.085 |
| **Chemotherapy Med** | 0.051 | -0.063 | -0.365 | 0.033 | -0.080 | 0.062 | -0.237 | -0.046 | 0.117 |
| **Immunosuppressive Med** | -0.099 | -0.078 | 0.003 | -0.399 | -0.009 | 0.137 | 0.162 | -0.251 | 0.142 |
| **Covid Infection** | -0.564 | -0.799 | -0.749 | -0.281 | -0.500 | -0.827 | -0.881 | -0.351 | 0.619 |
| **Covid Fever** | 0.935 | 0.076 | -0.343 | 0.156 | 0.253 | 0.043 | 0.119 | 0.334 | 0.282 |
| **Covid Fatigue** | -0.423 | 0.663 | 0.319 | 0.211 | 0.053 | 0.217 | 0.450 | -0.031 | 0.296 |
| **Covid Cough** | -0.105 | 0.025 | -0.200 | -0.055 | 0.102 | 0.113 | 0.073 | -0.019 | 0.086 |
| **Covid Gastrointestinal** | 0.074 | -0.016 | -0.205 | 0.172 | 0.107 | 0.299 | 0.039 | -0.265 | 0.147 |
| **Covid Anosmia** | -0.270 | 0.109 | -0.039 | -0.072 | -0.094 | 0.307 | 0.303 | 0.179 | 0.172 |
| **Covid RespiratoryProblems** | -0.252 | -0.198 | 0.015 | -0.053 | 0.105 | -0.087 | -0.131 | -0.093 | 0.117 |
| **Covid ConsciousnessDisorder** | 0.518 | 0.075 | 0.123 | -0.017 | 0.196 | 0.256 | 0.019 | 0.010 | 0.152 |
| **Covid Paresis** | -0.421 | 0.216 | -0.450 | 0.105 | -0.170 | -0.287 | -0.163 | 0.261 | 0.259 |
| **Covid ChestPain** | 0.414 | 0.137 | 0.233 | 0.388 | 0.195 | 0.463 | 0.401 | 0.293 | 0.316 |
| **Covid Headache** | 0.460 | 0.646 | 1.202 | 0.008 | 0.220 | 0.410 | 0.272 | 0.328 | 0.443 |
| **Covid SoreThroat** | -0.124 | -0.164 | -0.030 | -0.174 | -0.093 | 0.395 | 0.209 | 0.185 | 0.172 |
| **Covid Vertigo** | 0.445 | 0.052 | 0.449 | 0.526 | 0.398 | -0.041 | 0.168 | -0.073 | 0.269 |
| **Covid hospitalization** | -0.119 | 0.180 | 0.092 | -0.028 | 0.315 | -0.160 | 0.138 | -0.100 | 0.142 |
| **BMI** | 0.964 | 0.385 | 0.759 | -0.112 | -0.012 | 1.177 | 0.528 | -0.714 | 0.581 |

| **Sinopharm D2** | **Dose2_Fever** | **Dose2_Fatigue** | **Dose2_Headache** | **Dose2_Nausea** | **Dose2_Chills** | **Dose2_Joint_Pain** | **Dose2_Muscle_Pain** | **Dose2_ Local side effects** | **Avg** |
| --- | --- | --- | --- | --- | --- | --- | --- | --- | --- |
| **Sex** | -0.136 | -0.205 | -0.242 | -0.658 | -0.277 | -0.273 | -0.214 | -0.401 | 0.301 |
| **Age** | -0.150 | -0.097 | -0.127 | -0.876 | -0.103 | -0.067 | -0.125 | -0.320 | 0.233 |
| **A** | 0.079 | -0.001 | 0.010 | -0.591 | -0.059 | -0.122 | -0.016 | 0.038 | 0.115 |
| **AB** | -0.015 | -0.002 | 0.004 | -0.329 | 0.064 | 0.053 | 0.004 | 0.035 | 0.063 |
| **B** | -0.135 | 0.054 | 0.003 | -0.506 | -0.061 | 0.146 | 0.045 | -0.023 | 0.122 |
| **O** | 0.070 | -0.051 | -0.017 | -0.531 | 0.056 | -0.077 | -0.034 | -0.051 | 0.111 |
| **Smoking** | -0.069 | 0.071 | -0.001 | 0.091 | 0.077 | 0.165 | 0.019 | -0.062 | 0.069 |
| **Substance use** | 0.190 | 0.035 | 0.016 | 0.128 | 0.094 | -0.096 | -0.005 | -0.031 | 0.074 |
| **Alcohol dependancy** | 0.021 | 0.011 | -0.038 | 0.081 | 0.122 | -0.119 | -0.012 | 0.018 | 0.053 |
| **Background Diabetes** | 0.143 | -0.011 | -0.061 | -0.229 | 0.150 | 0.097 | 0.016 | -0.118 | 0.103 |
| **Background Cardiovascular disease** | 0.109 | 0.008 | -0.048 | -0.150 | 0.224 | -0.156 | 0.008 | -0.091 | 0.099 |
| **Background Hypertension** | -0.079 | -0.006 | -0.100 | -0.351 | -0.055 | 0.080 | 0.054 | -0.118 | 0.105 |
| **Background CancerPassive** | -0.029 | 0.005 | 0.015 | -0.087 | -0.114 | 0.095 | 0.013 | 0.042 | 0.050 |
| **Background CancerActive** | 0.049 | 0.041 | -0.007 | -0.034 | 0.240 | -0.008 | 0.035 | 0.026 | 0.055 |
| **Background Neurological** | -0.080 | 0.046 | 0.086 | -0.125 | -0.161 | 0.089 | 0.009 | 0.033 | 0.079 |
| **Background pulmonary** | 0.018 | 0.008 | 0.056 | -0.060 | 0.145 | -0.112 | 0.035 | 0.018 | 0.057 |
| **Background ImmuneSystem** | -0.052 | 0.038 | 0.037 | -0.080 | 0.137 | -0.242 | 0.031 | 0.071 | 0.086 |
| **Background hematologic** | -0.163 | -0.015 | -0.013 | -0.167 | -0.066 | 0.018 | -0.028 | 0.000 | 0.059 |
| **Background Gastrointestinal** | -0.058 | 0.038 | 0.067 | 0.022 | -0.162 | -0.125 | 0.015 | -0.047 | 0.067 |
| **Background Renal** | 0.180 | 0.025 | 0.007 | 0.200 | -0.073 | -0.017 | -0.013 | 0.042 | 0.070 |
| **Background Hepatic** | -0.061 | -0.001 | 0.013 | -0.224 | -0.050 | -0.183 | 0.024 | -0.013 | 0.071 |
| **Background Skeletal** | -0.074 | 0.027 | 0.016 | 0.021 | 0.188 | 0.522 | 0.089 | 0.023 | 0.120 |
| **Background Mental** | -0.043 | 0.051 | 0.019 | 0.151 | 0.107 | 0.089 | 0.046 | -0.008 | 0.064 |
| **Background Allergy** | 0.279 | 0.106 | 0.042 | -0.155 | 0.164 | 0.158 | 0.094 | 0.128 | 0.141 |
| **Background None** | -0.077 | -0.063 | -0.017 | -0.570 | -0.171 | -0.093 | -0.095 | -0.024 | 0.139 |
| **Hormonal med** | 0.196 | 0.062 | 0.031 | -0.001 | -0.088 | 0.341 | 0.022 | 0.062 | 0.100 |
| **Pregnancy** | 0.155 | 0.085 | 0.028 | -0.105 | 0.217 | 0.088 | 0.096 | 0.080 | 0.107 |
| **Respiratory Inhaler** | 0.003 | -0.003 | 0.029 | -0.047 | -0.114 | -0.004 | -0.019 | 0.005 | 0.028 |
| **Corticosteroid Med** | 0.090 | 0.006 | -0.030 | -0.052 | 0.011 | 0.012 | -0.005 | 0.028 | 0.029 |
| **Chemotherapy Med** | 0.032 | -0.012 | -0.059 | 0.126 | 0.021 | 0.051 | 0.034 | -0.053 | 0.049 |
| **Immunosuppressive Med** | -0.197 | -0.013 | 0.018 | -0.286 | -0.134 | -0.083 | 0.024 | 0.015 | 0.096 |
| **Covid Infection** | -0.136 | 0.019 | -0.001 | -0.157 | -0.066 | -0.020 | 0.019 | 0.013 | 0.054 |
| **Covid Fever** | 0.169 | 0.053 | 0.020 | 0.008 | 0.123 | 0.064 | 0.013 | 0.084 | 0.067 |
| **Covid Fatigue** | -0.077 | 0.078 | 0.075 | -0.042 | -0.072 | -0.073 | 0.084 | 0.009 | 0.064 |
| **Covid Cough** | 0.136 | -0.009 | 0.022 | 0.113 | 0.203 | 0.089 | -0.019 | 0.043 | 0.079 |
| **Covid Gastrointestinal** | -0.126 | 0.033 | 0.053 | 0.036 | 0.072 | -0.048 | 0.009 | -0.047 | 0.053 |
| **Covid Anosmia** | 0.170 | 0.066 | 0.003 | 0.055 | 0.108 | 0.369 | 0.088 | 0.039 | 0.112 |
| **Covid RespiratoryProblems** | 0.285 | -0.052 | 0.009 | -0.020 | 0.123 | -0.056 | -0.026 | 0.019 | 0.074 |
| **Covid ConsciousnessDisorder** | -0.131 | 0.019 | 0.006 | 0.020 | 0.148 | 0.240 | 0.028 | 0.005 | 0.075 |
| **Covid Paresis** | 0.228 | -0.005 | -0.002 | -0.080 | 0.061 | -0.104 | 0.011 | 0.012 | 0.063 |
| **Covid ChestPain** | 0.056 | 0.015 | 0.077 | 0.261 | -0.105 | 0.046 | 0.049 | 0.032 | 0.080 |
| **Covid Headache** | 0.154 | 0.091 | 0.184 | 0.002 | 0.141 | 0.099 | 0.094 | 0.025 | 0.099 |
| **Covid SoreThroat** | 0.445 | 0.023 | 0.028 | 0.035 | 0.166 | 0.052 | 0.006 | 0.089 | 0.106 |
| **Covid Vertigo** | -0.157 | 0.088 | 0.074 | -0.066 | -0.267 | -0.084 | 0.009 | 0.044 | 0.099 |
| **Covid hospitalization** | -0.031 | 0.048 | 0.015 | -0.203 | 0.016 | 0.179 | 0.028 | -0.012 | 0.067 |
| **Dose1_Fever** | 3.103 | 0.287 | 0.310 | 0.049 | 0.833 | 0.244 | 0.265 | 0.150 | 0.655 |
| **Dose1_Fatigue** | 0.432 | 1.663 | 0.503 | 0.223 | 0.235 | 0.504 | 0.584 | 0.283 | 0.553 |
| **Dose1_Headache** | 0.243 | 0.427 | 1.407 | 0.240 | 0.269 | 0.364 | 0.278 | 0.152 | 0.423 |
| **Dose1_Nausea** | -0.013 | 0.122 | 0.082 | 2.541 | 0.048 | 0.274 | 0.126 | -0.028 | 0.404 |
| **Dose1_Chills** | 0.240 | 0.122 | 0.103 | 0.332 | 2.291 | -0.083 | 0.099 | 0.037 | 0.413 |
| **Dose1_Joint_Pain** | 0.098 | 0.272 | 0.249 | 0.196 | 0.197 | 2.420 | 0.591 | 0.052 | 0.509 |
| **Dose1_Muscle_Pain** | 0.236 | 0.352 | 0.297 | 0.233 | 0.178 | 0.947 | 1.196 | 0.216 | 0.457 |
| **Dose1_ Local side effects** | 0.238 | 0.137 | 0.124 | -0.301 | 0.102 | -0.067 | 0.253 | 1.710 | 0.367 |
| **BMI** | 0.048 | 0.012 | 0.014 | -0.394 | 0.106 | -0.005 | 0.013 | -0.039 | 0.079 |

| **Covaxin D1** | **Dose1_Fever** | **Dose1_Fatigue** | **Dose1_Headache** | **Dose1_Nausea** | **Dose1_Chills** | **Dose1_Joint_Pain** | **Dose1_Muscle_Pain** | **Dose1_ Local side effects** | **Avg** |
| --- | --- | --- | --- | --- | --- | --- | --- | --- | --- |
| **Sex** | -0.173 | -0.338 | -0.322 | -0.383 | -0.016 | -0.084 | -0.195 | -0.499 | 0.251 |
| **Age** | -0.410 | -0.492 | -0.093 | -1.201 | -0.010 | -0.780 | -0.795 | -0.377 | 0.520 |
| **A** | -0.059 | -0.018 | -0.053 | -0.336 | 0.033 | -0.155 | -0.059 | -0.093 | 0.101 |
| **AB** | -0.207 | -0.060 | -0.087 | -0.099 | -0.018 | 0.133 | 0.052 | -0.169 | 0.103 |
| **B** | -0.137 | 0.145 | 0.176 | -0.647 | 0.010 | -0.571 | -0.211 | 0.134 | 0.254 |
| **O** | -0.354 | 0.151 | -0.036 | -0.577 | -0.025 | -0.156 | -0.099 | 0.128 | 0.191 |
| **Smoking** | 0.411 | 1.009 | 0.273 | 0.892 | 0.018 | 0.200 | 0.489 | -0.038 | 0.416 |
| **Substance use** | 0.021 | 0.031 | 0.100 | 0.151 | 0.006 | 0.341 | 0.097 | -0.008 | 0.094 |
| **Alcohol dependancy** | 0.115 | -0.015 | 0.086 | 0.070 | 0.002 | 0.315 | 0.036 | 0.066 | 0.088 |
| **Background Diabetes** | -0.251 | -0.378 | -0.159 | -0.020 | -0.003 | 0.013 | -0.047 | 0.183 | 0.132 |
| **Background Cardiovascular disease** | 0.088 | 0.160 | -0.009 | 0.005 | 0.027 | 0.207 | -0.015 | -0.066 | 0.072 |
| **Background Hypertension** | 0.055 | 0.138 | 0.130 | 0.067 | 0.013 | 0.221 | 0.204 | -0.035 | 0.108 |
| **Background CancerPassive** | -0.123 | -0.390 | -0.061 | -0.206 | -0.008 | -0.499 | -0.569 | -0.123 | 0.247 |
| **Background CancerActive** | -0.019 | -0.116 | -0.024 | -0.049 | -0.001 | -0.100 | -0.127 | 0.029 | 0.058 |
| **Background Neurological** | 0.056 | -0.146 | 0.091 | -0.568 | 0.011 | -0.082 | 0.063 | 0.090 | 0.138 |
| **Background pulmonary** | 0.184 | 0.303 | -0.029 | -0.226 | 0.017 | 0.245 | 0.441 | 0.177 | 0.203 |
| **Background ImmuneSystem** | -0.086 | 0.149 | -0.032 | 0.015 | 0.011 | -0.164 | 0.033 | 0.101 | 0.074 |
| **Background hematologic** | -0.055 | 0.524 | 0.013 | -0.307 | 0.001 | -0.146 | 0.207 | 0.052 | 0.163 |
| **Background Gastrointestinal** | 0.046 | -0.397 | -0.060 | -0.937 | 0.037 | 0.183 | -0.080 | -0.091 | 0.229 |
| **Background Renal** | 0.017 | 0.152 | 0.073 | 0.319 | 0.010 | -0.270 | -0.228 | -0.107 | 0.147 |
| **Background Hepatic** | -0.074 | -0.069 | -0.120 | -0.314 | -0.001 | -0.242 | -0.270 | -0.156 | 0.156 |
| **Background Skeletal** | -0.011 | 0.022 | 0.316 | 0.554 | 0.017 | 0.215 | 0.443 | 0.066 | 0.205 |
| **Background Mental** | -0.213 | -0.110 | -0.022 | -0.700 | 0.001 | -0.432 | -0.535 | 0.252 | 0.283 |
| **Background Allergy** | 0.025 | 0.038 | 0.192 | 0.269 | 0.005 | -0.127 | 0.279 | 0.019 | 0.119 |
| **Background None** | -0.323 | 0.056 | 0.104 | 0.283 | -0.030 | 0.008 | 0.202 | -0.136 | 0.143 |
| **Hormonal med** | 0.180 | 0.689 | 0.350 | 0.993 | 0.043 | 0.577 | 0.052 | -0.025 | 0.364 |
| **Pregnancy** | 0.043 | -0.073 | -0.046 | -0.400 | 0.013 | 0.566 | 0.252 | -0.001 | 0.174 |
| **Respiratory Inhaler** | 0.184 | 0.143 | 0.373 | -0.305 | 0.034 | 0.492 | 0.217 | 0.018 | 0.221 |
| **Corticosteroid Med** | -0.009 | 0.754 | 0.199 | 0.410 | 0.040 | -0.199 | -0.013 | -0.136 | 0.220 |
| **Chemotherapy Med** | -0.052 | -0.532 | -0.131 | -0.066 | 0.017 | 0.063 | -0.146 | -0.171 | 0.147 |
| **Immunosuppressive Med** | 0.017 | -0.442 | 0.048 | -0.533 | 0.024 | -0.219 | 0.221 | -0.197 | 0.213 |
| **Covid Infection** | -0.560 | -1.222 | -0.374 | -1.150 | -0.038 | -0.416 | -0.961 | -0.105 | 0.603 |
| **Covid Fever** | 0.070 | 0.286 | 0.145 | 0.062 | 0.038 | 0.476 | 0.254 | 0.229 | 0.195 |
| **Covid Fatigue** | -0.070 | 0.528 | -0.164 | -0.024 | 0.006 | -0.158 | 0.470 | 0.236 | 0.207 |
| **Covid Cough** | 0.217 | 0.206 | 0.244 | 0.269 | 0.022 | 0.040 | 0.141 | 0.122 | 0.158 |
| **Covid Gastrointestinal** | 0.122 | -0.038 | -0.090 | 0.685 | 0.021 | 0.354 | 0.180 | -0.153 | 0.205 |
| **Covid Anosmia** | -0.129 | 0.141 | 0.069 | 0.104 | 0.022 | 0.134 | -0.024 | 0.144 | 0.096 |
| **Covid RespiratoryProblems** | 0.163 | 0.203 | -0.088 | 0.578 | 0.014 | -0.683 | -0.116 | -0.090 | 0.242 |
| **Covid ConsciousnessDisorder** | 0.179 | 0.190 | 0.084 | 0.960 | 0.022 | 0.476 | 0.399 | 0.031 | 0.293 |
| **Covid Paresis** | 0.095 | 0.241 | 0.044 | 0.519 | 0.014 | 0.461 | 0.217 | -0.089 | 0.210 |
| **Covid ChestPain** | 0.129 | 0.159 | 0.054 | 0.167 | 0.047 | 0.426 | 0.698 | -0.122 | 0.225 |
| **Covid Headache** | 0.081 | -0.034 | 0.403 | -0.434 | 0.009 | 0.132 | 0.123 | 0.130 | 0.168 |
| **Covid SoreThroat** | 0.150 | 0.102 | 0.184 | 0.109 | 0.032 | 0.131 | 0.335 | 0.013 | 0.132 |
| **Covid Vertigo** | 0.152 | -0.048 | 0.048 | 0.518 | 0.034 | -0.047 | -0.027 | -0.078 | 0.119 |
| **Covid hospitalization** | 0.260 | 0.475 | 0.072 | 0.723 | 0.010 | 0.735 | 0.741 | -0.172 | 0.398 |
| **BMI** | -0.307 | -1.142 | -0.079 | -0.549 | -0.009 | -0.502 | -0.568 | -0.217 | 0.421 |

| **Covaxin D2** | **Dose2_Fever** | **Dose2_Fatigue** | **Dose2_Headache** | **Dose2_Nausea** | **Dose2_Chills** | **Dose2_Joint_Pain** | **Dose2_Muscle_Pain** | **Dose2_ Local side effects** | **Avg** |
| --- | --- | --- | --- | --- | --- | --- | --- | --- | --- |
| **Sex** | -0.154 | -0.298 | -0.338 | -0.569 | -0.439 | -0.103 | -0.186 | -0.201 | 0.286 |
| **Age** | -0.765 | -0.172 | -0.119 | -0.769 | -0.335 | -0.414 | -0.462 | -0.101 | 0.392 |
| **A** | -0.419 | -0.009 | 0.110 | -0.683 | -0.436 | -0.253 | -0.126 | 0.028 | 0.258 |
| **AB** | -0.131 | 0.190 | 0.137 | 0.172 | 0.009 | 0.057 | -0.162 | -0.042 | 0.113 |
| **B** | -0.416 | -0.181 | -0.193 | -0.627 | -0.236 | -0.403 | -0.360 | 0.049 | 0.308 |
| **O** | -0.360 | 0.000 | -0.054 | -0.601 | -0.270 | -0.345 | -0.141 | -0.035 | 0.226 |
| **Smoking** | -0.511 | 0.126 | 0.058 | -0.762 | -0.150 | -0.185 | -0.116 | 0.034 | 0.243 |
| **Substance use** | -0.060 | 0.098 | 0.122 | -0.069 | -0.027 | 0.148 | 0.095 | -0.007 | 0.078 |
| **Alcohol dependancy** | 0.173 | 0.086 | 0.035 | -0.026 | -0.024 | 0.016 | -0.008 | 0.024 | 0.049 |
| **Background Diabetes** | -0.535 | -0.255 | -0.028 | 0.049 | -0.118 | -0.127 | -0.058 | -0.144 | 0.164 |
| **Background Cardiovascular disease** | 0.377 | 0.131 | 0.122 | -0.168 | 0.156 | -0.120 | -0.046 | 0.071 | 0.149 |
| **Background Hypertension** | -0.097 | -0.008 | -0.225 | -0.118 | 0.026 | -0.119 | -0.138 | -0.079 | 0.101 |
| **Background CancerPassive** | 0.170 | 0.054 | 0.097 | 0.257 | 0.018 | 0.029 | -0.061 | -0.086 | 0.096 |
| **Background CancerActive** | 0.401 | 0.061 | 0.079 | -0.045 | 0.078 | -0.019 | -0.013 | -0.067 | 0.095 |
| **Background Neurological** | 0.324 | 0.156 | 0.100 | -0.181 | 0.104 | 0.154 | 0.253 | -0.043 | 0.164 |
| **Background pulmonary** | 0.078 | -0.009 | -0.105 | -0.284 | -0.048 | -0.035 | 0.022 | -0.003 | 0.073 |
| **Background ImmuneSystem** | 0.185 | 0.063 | 0.077 | -0.398 | 0.140 | -0.109 | -0.208 | -0.037 | 0.152 |
| **Background hematologic** | -0.123 | -0.080 | -0.034 | -0.034 | -0.026 | -0.033 | -0.045 | -0.039 | 0.052 |
| **Background Gastrointestinal** | -0.176 | -0.013 | 0.041 | -0.073 | 0.126 | -0.141 | -0.007 | 0.019 | 0.075 |
| **Background Renal** | 0.073 | -0.088 | 0.073 | 0.010 | -0.014 | -0.084 | -0.186 | -0.105 | 0.079 |
| **Background Hepatic** | 0.221 | -0.015 | -0.024 | -0.132 | -0.095 | -0.113 | -0.138 | -0.213 | 0.119 |
| **Background Skeletal** | -0.633 | -0.049 | -0.070 | -0.105 | -0.170 | 0.047 | -0.035 | -0.079 | 0.149 |
| **Background Mental** | -0.459 | -0.115 | -0.006 | 0.132 | -0.124 | -0.138 | -0.166 | -0.045 | 0.148 |
| **Background Allergy** | -0.580 | 0.309 | 0.176 | -0.181 | -0.187 | 0.005 | 0.037 | 0.345 | 0.227 |
| **Background None** | -0.838 | -0.277 | -0.200 | -0.486 | -0.607 | -0.612 | -0.404 | 0.053 | 0.435 |
| **Hormonal med** | 0.399 | 0.184 | 0.289 | -0.091 | 0.079 | 0.106 | 0.023 | -0.157 | 0.166 |
| **Pregnancy** | -0.211 | 0.077 | -0.008 | 0.418 | -0.066 | 0.010 | -0.184 | -0.124 | 0.137 |
| **Respiratory Inhaler** | -0.119 | 0.158 | -0.107 | -0.197 | -0.111 | 0.109 | 0.089 | -0.076 | 0.121 |
| **Corticosteroid Med** | -0.269 | -0.142 | -0.023 | 0.038 | -0.150 | -0.224 | -0.310 | -0.075 | 0.154 |
| **Chemotherapy Med** | 0.286 | 0.047 | 0.091 | 0.060 | 0.035 | 0.047 | -0.056 | -0.068 | 0.086 |
| **Immunosuppressive Med** | -0.395 | -0.228 | 0.068 | -0.318 | -0.085 | -0.243 | -0.194 | -0.042 | 0.197 |
| **Covid Infection** | -0.576 | -0.145 | 0.059 | -0.177 | -0.070 | -0.087 | -0.135 | -0.215 | 0.183 |
| **Covid Fever** | 0.091 | 0.104 | 0.094 | 0.039 | 0.165 | 0.087 | 0.003 | 0.082 | 0.083 |
| **Covid Fatigue** | 0.352 | 0.320 | 0.373 | 0.087 | 0.028 | 0.169 | 0.267 | 0.110 | 0.213 |
| **Covid Cough** | -0.050 | 0.014 | -0.056 | 0.115 | -0.168 | -0.205 | -0.070 | -0.126 | 0.101 |
| **Covid Gastrointestinal** | 0.404 | 0.074 | -0.076 | -0.131 | 0.070 | -0.047 | 0.143 | -0.076 | 0.128 |
| **Covid Anosmia** | -0.389 | 0.125 | -0.055 | 0.260 | -0.060 | -0.199 | 0.089 | 0.062 | 0.155 |
| **Covid RespiratoryProblems** | -0.155 | 0.069 | -0.177 | 0.119 | -0.096 | -0.009 | -0.207 | 0.072 | 0.113 |
| **Covid ConsciousnessDisorder** | 0.376 | -0.002 | 0.056 | 0.169 | 0.145 | 0.079 | -0.026 | 0.089 | 0.118 |
| **Covid Paresis** | -0.239 | 0.044 | 0.101 | 0.187 | -0.034 | 0.149 | 0.074 | -0.023 | 0.106 |
| **Covid ChestPain** | 0.053 | -0.018 | 0.297 | 0.016 | 0.212 | 0.214 | 0.178 | -0.087 | 0.134 |
| **Covid Headache** | 0.139 | -0.213 | 0.260 | 0.365 | 0.048 | 0.133 | 0.116 | 0.372 | 0.206 |
| **Covid SoreThroat** | 0.141 | 0.081 | -0.018 | 0.052 | 0.005 | 0.014 | -0.130 | 0.289 | 0.091 |
| **Covid Vertigo** | 0.413 | 0.252 | 0.197 | 0.080 | 0.243 | 0.224 | 0.345 | -0.056 | 0.226 |
| **Covid hospitalization** | -0.298 | 0.226 | 0.223 | -0.175 | -0.066 | 0.220 | 0.415 | 0.120 | 0.218 |
| **Dose1_Fever** | 2.854 | 0.255 | 0.496 | 0.274 | 0.372 | 0.177 | 0.408 | 0.178 | 0.627 |
| **Dose1_Fatigue** | 0.171 | 1.747 | 0.368 | 0.182 | -0.142 | 0.029 | 0.211 | 0.319 | 0.396 |
| **Dose1_Headache** | 0.252 | 0.139 | 1.475 | 0.300 | 0.035 | 0.074 | 0.083 | 0.148 | 0.313 |
| **Dose1_Nausea** | 0.450 | 0.220 | 0.314 | 2.898 | 0.287 | 0.278 | 0.361 | 0.148 | 0.620 |
| **Dose1_Chills** | 0.271 | -0.062 | 0.076 | 0.250 | 1.565 | 0.016 | 0.114 | 0.024 | 0.297 |
| **Dose1_Joint_Pain** | -0.220 | 0.192 | -0.015 | -0.095 | -0.143 | 1.671 | 0.504 | -0.043 | 0.360 |
| **Dose1_Muscle_Pain** | 0.127 | 0.426 | 0.274 | 0.139 | 0.042 | 0.507 | 1.623 | 0.333 | 0.434 |
| **Dose1_ Local side effects** | 0.308 | 0.132 | 0.106 | -0.381 | -0.168 | -0.279 | -0.146 | 1.666 | 0.398 |
| **BMI** | -0.451 | -0.089 | -0.001 | -0.252 | -0.213 | -0.152 | -0.186 | -0.016 | 0.170 |

| **Pfizer D1** | **Dose1_Fever** | **Dose1_Fatigue** | **Dose1_Headache** | **Dose1_Nausea** | **Dose1_Chills** | **Dose1_Joint_Pain** | **Dose1_Muscle_Pain** | **Dose1_ Local side effects** | **Avg** |
| --- | --- | --- | --- | --- | --- | --- | --- | --- | --- |
| **Sex** | -0.446 | -0.871 | -1.023 | -0.147 | -1.082 | -0.028 | 0.040 | -0.705 | 0.543 |
| **Age** | -0.881 | -1.282 | -0.489 | -0.186 | -1.141 | 0.006 | -0.472 | -0.586 | 0.630 |
| **A** | 0.251 | 0.362 | 0.039 | -0.149 | 0.212 | 0.030 | 0.247 | 0.535 | 0.228 |
| **AB** | -0.502 | 0.303 | -0.007 | -0.025 | 0.775 | -0.011 | -0.521 | -0.388 | 0.316 |
| **B** | 0.122 | 0.470 | -0.010 | -0.035 | 0.653 | -0.010 | 0.247 | 0.519 | 0.258 |
| **O** | 0.051 | 0.169 | 0.350 | -0.145 | 0.844 | -0.006 | 0.273 | 0.635 | 0.309 |
| **Smoking** | 0.433 | 0.527 | 0.411 | -0.110 | 0.804 | 0.035 | 0.694 | -0.191 | 0.401 |
| **Substance use** | 0.000 | 0.000 | 0.000 | 0.000 | 0.000 | 0.000 | 0.000 | 0.000 | 0.000 |
| **Alcohol dependancy** | 0.597 | 1.088 | 0.279 | 0.005 | 3.140 | 0.007 | 0.309 | -1.051 | 0.810 |
| **Background Diabetes** | -0.415 | 0.387 | -0.380 | -0.005 | 2.341 | -0.007 | 0.285 | 0.134 | 0.494 |
| **Background Cardiovascular disease** | 0.000 | 0.000 | 0.000 | 0.000 | 0.000 | 0.000 | 0.000 | 0.000 | 0.000 |
| **Background Hypertension** | -0.318 | 0.462 | -0.557 | -0.032 | 1.152 | 0.009 | 0.643 | -0.111 | 0.411 |
| **Background CancerPassive** | 0.000 | 0.000 | 0.000 | 0.000 | 0.000 | 0.000 | 0.000 | 0.000 | 0.000 |
| **Background CancerActive** | 0.000 | 0.000 | 0.000 | 0.000 | 0.000 | 0.000 | 0.000 | 0.000 | 0.000 |
| **Background Neurological** | -0.269 | 0.970 | 0.098 | -0.007 | -0.476 | -0.003 | -0.418 | 0.126 | 0.296 |
| **Background pulmonary** | -0.377 | 0.630 | -0.350 | -0.025 | -1.552 | 0.002 | -0.888 | 0.148 | 0.496 |
| **Background ImmuneSystem** | 0.309 | 1.928 | -0.009 | -0.015 | -0.675 | -0.007 | -0.048 | -0.749 | 0.467 |
| **Background hematologic** | 0.000 | 0.000 | 0.000 | 0.000 | 0.000 | 0.000 | 0.000 | 0.000 | 0.000 |
| **Background Gastrointestinal** | 0.616 | -1.211 | 0.243 | -0.006 | -0.290 | -0.003 | -0.359 | -0.534 | 0.408 |
| **Background Renal** | -0.198 | -1.231 | -0.208 | -0.003 | -0.491 | -0.001 | 0.753 | 0.080 | 0.371 |
| **Background Hepatic** | -0.039 | 1.735 | -0.063 | -0.003 | 1.249 | -0.001 | 0.390 | 0.061 | 0.443 |
| **Background Skeletal** | 0.000 | 0.000 | 0.000 | 0.000 | 0.000 | 0.000 | 0.000 | 0.000 | 0.000 |
| **Background Mental** | 0.385 | 4.006 | 0.300 | -0.009 | -0.471 | -0.004 | 0.424 | 0.338 | 0.742 |
| **Background Allergy** | -0.743 | 0.176 | -0.095 | -0.123 | 1.541 | 0.001 | 1.131 | 0.433 | 0.530 |
| **Background None** | -0.525 | 0.622 | -0.515 | -0.223 | 1.162 | 0.016 | 0.419 | -0.072 | 0.444 |
| **Hormonal med** | -0.021 | 0.617 | 0.550 | -0.069 | 0.665 | 0.038 | -0.080 | 0.297 | 0.292 |
| **Pregnancy** | -0.481 | 0.348 | -0.496 | -0.096 | 0.130 | -0.011 | -0.558 | -0.107 | 0.278 |
| **Respiratory Inhaler** | -0.397 | 0.482 | -0.160 | -0.071 | 0.955 | 0.003 | -0.293 | -0.397 | 0.345 |
| **Corticosteroid Med** | 0.193 | 0.824 | 0.004 | -0.044 | -1.591 | 0.013 | 0.570 | 0.056 | 0.412 |
| **Chemotherapy Med** | 0.389 | 0.643 | 0.182 | -0.036 | 0.823 | 0.003 | 0.042 | 0.049 | 0.271 |
| **Immunosuppressive Med** | 0.156 | -2.111 | -0.005 | -0.042 | 0.291 | 0.000 | -0.351 | -0.325 | 0.410 |
| **Covid Infection** | -0.730 | -0.122 | -0.177 | -0.056 | -3.015 | 0.006 | -0.869 | -0.063 | 0.630 |
| **Covid Fever** | 0.597 | 0.150 | 0.535 | -0.014 | -0.271 | 0.029 | -0.070 | 0.040 | 0.213 |
| **Covid Fatigue** | -0.090 | 0.535 | 0.053 | -0.046 | 0.206 | 0.002 | -0.637 | -0.053 | 0.203 |
| **Covid Cough** | 0.327 | 1.344 | 0.405 | -0.017 | 0.155 | 0.004 | 0.021 | -0.598 | 0.359 |
| **Covid Gastrointestinal** | 0.586 | -0.063 | 0.346 | 0.007 | 4.247 | 0.013 | 1.115 | -1.112 | 0.936 |
| **Covid Anosmia** | 0.269 | -1.045 | -0.329 | -0.025 | -1.789 | 0.009 | 1.002 | 0.406 | 0.609 |
| **Covid RespiratoryProblems** | 0.227 | -1.791 | -0.087 | 0.003 | 0.367 | 0.006 | -0.063 | -0.200 | 0.343 |
| **Covid ConsciousnessDisorder** | 0.000 | 0.000 | 0.000 | 0.000 | 0.000 | 0.000 | 0.000 | 0.000 | 0.000 |
| **Covid Paresis** | 0.000 | 0.000 | 0.000 | 0.000 | 0.000 | 0.000 | 0.000 | 0.000 | 0.000 |
| **Covid ChestPain** | 1.166 | 1.350 | 0.423 | 0.001 | 1.981 | 0.014 | 0.483 | 0.069 | 0.686 |
| **Covid Headache** | 0.108 | 1.435 | 0.525 | -0.013 | 1.530 | 0.020 | 0.474 | 0.001 | 0.513 |
| **Covid SoreThroat** | 0.438 | -1.657 | 0.169 | -0.015 | 0.524 | 0.005 | -0.290 | 0.414 | 0.439 |
| **Covid Vertigo** | -0.039 | 0.221 | 0.348 | 0.008 | 2.071 | 0.022 | 0.246 | -0.031 | 0.373 |
| **Covid hospitalization** | 0.353 | 0.772 | 0.112 | 0.008 | 0.733 | 0.008 | 0.285 | 0.209 | 0.310 |
| **BMI** | -0.422 | 0.075 | 0.231 | -0.128 | -0.906 | 0.015 | 0.427 | -0.577 | 0.348 |

| **Pfizer D2** | **Dose2_Fever** | **Dose2_Fatigue** | **Dose2_Headache** | **Dose2_Nausea** | **Dose2_Chills** | **Dose2_Joint_Pain** | **Dose2_Muscle_Pain** | **Dose2_ Local side effects** | **Avg** |
| --- | --- | --- | --- | --- | --- | --- | --- | --- | --- |
| **Sex** | -0.404 | -0.355 | -0.247 | -0.914 | -0.508 | -0.460 | -0.693 | 0.404 | 0.498 |
| **Age** | -0.134 | -0.091 | -0.600 | -0.630 | -0.364 | -0.240 | 0.062 | 0.419 | 0.317 |
| **A** | -0.208 | -0.014 | -0.013 | -0.736 | -0.298 | -0.374 | -0.365 | 0.089 | 0.262 |
| **AB** | -0.145 | -0.193 | -0.187 | -0.409 | -0.150 | -0.131 | -0.341 | 0.225 | 0.223 |
| **B** | 0.126 | 0.094 | 0.595 | -0.073 | -0.063 | -0.049 | 0.214 | 0.549 | 0.220 |
| **O** | 0.242 | 0.226 | 0.221 | 0.017 | -0.007 | -0.025 | 0.119 | -0.774 | 0.204 |
| **Smoking** | -0.006 | -0.064 | 0.486 | -0.395 | -0.151 | -0.037 | -1.056 | 0.039 | 0.279 |
| **Substance use** | 0.000 | 0.000 | 0.000 | 0.000 | 0.000 | 0.000 | 0.000 | 0.000 | 0.000 |
| **Alcohol dependancy** | -0.016 | -0.070 | -0.463 | -0.083 | -0.023 | -0.039 | -0.882 | -0.705 | 0.285 |
| **Background Diabetes** | 0.038 | -0.087 | -0.677 | -0.201 | -0.088 | 0.017 | -2.317 | 0.356 | 0.473 |
| **Background Cardiovascular disease** | 0.000 | 0.000 | 0.000 | 0.000 | 0.000 | 0.000 | 0.000 | 0.000 | 0.000 |
| **Background Hypertension** | 0.012 | 0.160 | -0.329 | -0.187 | -0.091 | 0.126 | 0.245 | 0.227 | 0.172 |
| **Background CancerPassive** | 0.000 | 0.000 | 0.000 | 0.000 | 0.000 | 0.000 | 0.000 | 0.000 | 0.000 |
| **Background CancerActive** | 0.000 | 0.000 | 0.000 | 0.000 | 0.000 | 0.000 | 0.000 | 0.000 | 0.000 |
| **Background Neurological** | -0.023 | -0.054 | -0.309 | -0.096 | -0.032 | -0.028 | -1.079 | 0.265 | 0.236 |
| **Background pulmonary** | -0.132 | -0.182 | -0.219 | -0.362 | -0.193 | -0.096 | -2.164 | -0.535 | 0.485 |
| **Background ImmuneSystem** | 0.011 | -0.047 | 0.055 | -0.194 | -0.005 | 0.074 | 1.030 | 0.540 | 0.245 |
| **Background hematologic** | 0.000 | 0.000 | 0.000 | 0.000 | 0.000 | 0.000 | 0.000 | 0.000 | 0.000 |
| **Background Gastrointestinal** | 0.045 | 0.051 | 0.449 | -0.132 | 0.027 | -0.063 | 0.302 | 0.748 | 0.227 |
| **Background Renal** | 0.074 | 0.043 | 0.356 | 0.360 | 0.067 | -0.030 | 1.333 | -0.642 | 0.363 |
| **Background Hepatic** | 0.000 | 0.000 | 0.000 | 0.000 | 0.000 | 0.000 | 0.000 | 0.000 | 0.000 |
| **Background Skeletal** | 0.000 | 0.000 | 0.000 | 0.000 | 0.000 | 0.000 | 0.000 | 0.000 | 0.000 |
| **Background Mental** | 0.038 | 0.045 | 0.249 | -0.067 | -0.043 | 0.038 | 2.178 | 0.094 | 0.344 |
| **Background Allergy** | -0.039 | -0.009 | 0.159 | 0.065 | 0.152 | -0.234 | 0.109 | -0.112 | 0.110 |
| **Background None** | -0.098 | 0.026 | -0.704 | -0.662 | -0.414 | -0.305 | 0.403 | -0.345 | 0.370 |
| **Hormonal med** | 0.094 | 0.110 | -0.173 | -0.139 | 0.448 | 0.400 | 0.452 | 0.419 | 0.279 |
| **Pregnancy** | -0.169 | -0.036 | -0.334 | -0.630 | -0.270 | -0.235 | -0.428 | -0.619 | 0.340 |
| **Respiratory Inhaler** | -0.252 | -0.165 | -0.853 | 0.059 | -0.220 | -0.069 | -0.579 | 0.538 | 0.342 |
| **Corticosteroid Med** | 0.063 | 0.078 | -0.026 | 0.071 | 0.008 | 0.130 | 1.510 | 0.545 | 0.304 |
| **Chemotherapy Med** | -0.015 | 0.017 | 0.311 | -0.083 | -0.054 | -0.012 | -2.165 | 0.136 | 0.349 |
| **Immunosuppressive Med** | 0.019 | 0.052 | 0.048 | -0.187 | -0.025 | 0.005 | 0.820 | -0.013 | 0.146 |
| **Covid Infection** | 0.006 | 0.068 | 0.210 | -0.111 | -0.015 | -0.024 | 0.095 | 0.299 | 0.103 |
| **Covid Fever** | 0.000 | 0.000 | 0.000 | 0.000 | 0.000 | 0.000 | 0.000 | 0.000 | 0.000 |
| **Covid Fatigue** | 0.006 | 0.068 | 0.210 | -0.111 | -0.015 | -0.024 | 0.095 | 0.299 | 0.103 |
| **Covid Cough** | 0.000 | 0.000 | 0.000 | 0.000 | 0.000 | 0.000 | 0.000 | 0.000 | 0.000 |
| **Covid Gastrointestinal** | 0.000 | 0.000 | 0.000 | 0.000 | 0.000 | 0.000 | 0.000 | 0.000 | 0.000 |
| **Covid Anosmia** | 0.062 | 0.070 | 0.088 | -0.046 | 0.049 | 0.052 | 1.075 | 0.094 | 0.192 |
| **Covid RespiratoryProblems** | 0.000 | 0.000 | 0.000 | 0.000 | 0.000 | 0.000 | 0.000 | 0.000 | 0.000 |
| **Covid ConsciousnessDisorder** | 0.000 | 0.000 | 0.000 | 0.000 | 0.000 | 0.000 | 0.000 | 0.000 | 0.000 |
| **Covid Paresis** | 0.000 | 0.000 | 0.000 | 0.000 | 0.000 | 0.000 | 0.000 | 0.000 | 0.000 |
| **Covid ChestPain** | 0.000 | 0.000 | 0.000 | 0.000 | 0.000 | 0.000 | 0.000 | 0.000 | 0.000 |
| **Covid Headache** | 0.075 | 0.037 | 0.372 | -0.039 | 0.063 | 0.067 | 0.327 | 0.053 | 0.129 |
| **Covid SoreThroat** | 0.055 | 0.062 | 0.513 | -0.058 | 0.047 | 0.042 | 1.906 | 0.068 | 0.344 |
| **Covid Vertigo** | 0.000 | 0.000 | 0.000 | 0.000 | 0.000 | 0.000 | 0.000 | 0.000 | 0.000 |
| **Covid hospitalization** | 0.000 | 0.000 | 0.000 | 0.000 | 0.000 | 0.000 | 0.000 | 0.000 | 0.000 |
| **Dose1_Fever** | 0.427 | 0.015 | -0.173 | -0.258 | -0.013 | -0.002 | -0.107 | 0.133 | 0.141 |
| **Dose1_Fatigue** | 0.057 | 0.768 | 0.045 | -0.648 | -0.069 | 0.058 | -0.060 | 0.428 | 0.267 |
| **Dose1_Headache** | 0.226 | 0.293 | 1.886 | 0.220 | -0.058 | 0.137 | -0.599 | 0.644 | 0.508 |
| **Dose1_Nausea** | -0.081 | -0.163 | -0.678 | 0.937 | 0.030 | 0.013 | -0.999 | 0.112 | 0.377 |
| **Dose1_Chills** | -0.099 | -0.043 | 0.725 | 0.540 | 0.258 | -0.030 | -0.339 | 0.346 | 0.297 |
| **Dose1_Joint_Pain** | 0.133 | 0.374 | -0.096 | 1.019 | 0.376 | 0.677 | 0.934 | 0.204 | 0.477 |
| **Dose1_Muscle_Pain** | 0.118 | 0.119 | 0.244 | 0.431 | 0.078 | 0.180 | 2.704 | 0.417 | 0.536 |
| **Dose1_ Local side effects** | 0.325 | 0.099 | 0.529 | -0.142 | -0.115 | -0.295 | 0.204 | 3.158 | 0.608 |
| **BMI** | 0.030 | -0.025 | -0.496 | -0.578 | -0.202 | -0.025 | 1.749 | -0.002 | 0.388 |

| **Moderna D1** | **Dose1_Fever** | **Dose1_Fatigue** | **Dose1_Headache** | **Dose1_Nausea** | **Dose1_Chills** | **Dose1_Joint_Pain** | **Dose1_Muscle_Pain** | **Dose1_ Local side effects** | **Avg** |
| --- | --- | --- | --- | --- | --- | --- | --- | --- | --- |
| **Sex** | -0.181 | -0.051 | 0.003 | -0.453 | -0.131 | -0.004 | -0.004 | -0.169 | 0.124 |
| **Age** | -0.295 | -0.029 | -0.003 | -0.406 | -0.325 | 0.005 | -0.005 | 0.003 | 0.134 |
| **A** | -0.419 | -0.011 | -0.018 | -0.412 | -0.358 | 0.040 | -0.008 | 0.006 | 0.159 |
| **AB** | -0.012 | -0.006 | -0.009 | -0.033 | -0.010 | -0.006 | -0.018 | 0.036 | 0.016 |
| **B** | -0.183 | -0.009 | -0.005 | -0.137 | -0.241 | -0.016 | -0.028 | 0.089 | 0.089 |
| **O** | -0.223 | -0.026 | 0.007 | -0.335 | -0.208 | -0.005 | 0.043 | -0.068 | 0.114 |
| **Smoking** | -0.374 | 0.026 | 0.016 | -0.162 | -0.070 | 0.024 | 0.012 | 0.128 | 0.101 |
| **Substance use** | 0.000 | 0.000 | 0.000 | 0.000 | 0.000 | 0.000 | 0.000 | 0.000 | 0.000 |
| **Alcohol dependancy** | -0.021 | -0.005 | -0.002 | -0.012 | -0.018 | -0.002 | -0.004 | 0.005 | 0.009 |
| **Background Diabetes** | -0.076 | -0.009 | -0.009 | -0.057 | 0.008 | -0.008 | -0.006 | 0.017 | 0.024 |
| **Background Cardiovascular disease** | 0.033 | -0.014 | -0.007 | -0.043 | -0.064 | -0.006 | -0.002 | 0.015 | 0.023 |
| **Background Hypertension** | 0.144 | 0.001 | 0.001 | -0.069 | 0.121 | 0.014 | 0.039 | -0.048 | 0.055 |
| **Background CancerPassive** | 0.000 | 0.000 | 0.000 | 0.000 | 0.000 | 0.000 | 0.000 | 0.000 | 0.000 |
| **Background CancerActive** | 0.000 | 0.000 | 0.000 | 0.000 | 0.000 | 0.000 | 0.000 | 0.000 | 0.000 |
| **Background Neurological** | -0.043 | 0.000 | -0.015 | -0.096 | -0.139 | -0.013 | -0.005 | -0.070 | 0.048 |
| **Background pulmonary** | 0.059 | 0.009 | 0.014 | 0.101 | 0.057 | 0.007 | 0.014 | 0.025 | 0.036 |
| **Background ImmuneSystem** | -0.018 | 0.004 | 0.002 | 0.028 | -0.003 | 0.003 | 0.016 | 0.013 | 0.011 |
| **Background hematologic** | -0.017 | 0.005 | -0.003 | -0.014 | -0.019 | 0.008 | 0.006 | 0.003 | 0.009 |
| **Background Gastrointestinal** | -0.101 | -0.009 | -0.014 | -0.080 | -0.115 | -0.003 | -0.005 | 0.023 | 0.044 |
| **Background Renal** | 0.025 | 0.000 | -0.006 | 0.052 | 0.024 | 0.005 | -0.008 | 0.010 | 0.016 |
| **Background Hepatic** | -0.015 | -0.005 | -0.002 | -0.016 | -0.021 | -0.002 | -0.004 | 0.004 | 0.009 |
| **Background Skeletal** | 0.000 | 0.000 | 0.000 | 0.000 | 0.000 | 0.000 | 0.000 | 0.000 | 0.000 |
| **Background Mental** | -0.096 | -0.001 | -0.010 | -0.072 | -0.022 | 0.001 | 0.003 | 0.017 | 0.028 |
| **Background Allergy** | -0.073 | 0.022 | 0.011 | -0.316 | -0.303 | -0.002 | 0.064 | 0.082 | 0.109 |
| **Background None** | -0.540 | -0.053 | 0.007 | -0.546 | -0.526 | -0.010 | -0.073 | -0.021 | 0.222 |
| **Hormonal med** | -0.015 | 0.000 | -0.002 | -0.096 | -0.113 | 0.003 | 0.016 | 0.066 | 0.039 |
| **Pregnancy** | -0.100 | -0.017 | -0.005 | -0.083 | 0.027 | -0.002 | -0.015 | 0.059 | 0.038 |
| **Respiratory Inhaler** | 0.158 | 0.048 | 0.043 | 0.093 | 0.077 | -0.003 | 0.042 | 0.091 | 0.069 |
| **Corticosteroid Med** | -0.025 | 0.039 | 0.014 | -0.035 | -0.113 | -0.024 | 0.010 | 0.076 | 0.042 |
| **Chemotherapy Med** | 0.063 | 0.035 | 0.022 | 0.010 | -0.036 | -0.017 | 0.013 | 0.062 | 0.032 |
| **Immunosuppressive Med** | 0.078 | 0.034 | 0.026 | 0.061 | -0.012 | -0.012 | 0.014 | 0.072 | 0.039 |
| **Covid Infection** | 0.268 | 0.085 | 0.094 | 0.170 | 0.182 | 0.053 | 0.026 | -0.262 | 0.143 |
| **Covid Fever** | 0.386 | 0.049 | 0.046 | 0.076 | 0.451 | 0.033 | 0.029 | 0.038 | 0.138 |
| **Covid Fatigue** | 0.353 | 0.086 | 0.095 | 0.225 | 0.268 | 0.052 | 0.043 | -0.185 | 0.163 |
| **Covid Cough** | 0.094 | 0.041 | 0.032 | 0.055 | 0.142 | 0.018 | 0.009 | -0.070 | 0.058 |
| **Covid Gastrointestinal** | 0.001 | 0.010 | 0.027 | 0.048 | 0.096 | -0.008 | 0.000 | 0.038 | 0.028 |
| **Covid Anosmia** | 0.231 | 0.065 | 0.073 | 0.017 | 0.133 | 0.023 | 0.012 | 0.051 | 0.076 |
| **Covid RespiratoryProblems** | 0.289 | 0.029 | 0.036 | 0.200 | 0.234 | 0.022 | 0.019 | 0.037 | 0.108 |
| **Covid ConsciousnessDisorder** | 0.000 | 0.000 | 0.000 | 0.000 | 0.000 | 0.000 | 0.000 | 0.000 | 0.000 |
| **Covid Paresis** | 0.059 | 0.003 | -0.009 | 0.031 | -0.028 | 0.013 | -0.003 | 0.018 | 0.021 |
| **Covid ChestPain** | 0.256 | 0.033 | 0.033 | 0.008 | 0.123 | 0.020 | 0.024 | 0.043 | 0.067 |
| **Covid Headache** | 0.227 | 0.074 | 0.096 | 0.167 | 0.232 | 0.048 | 0.033 | 0.066 | 0.118 |
| **Covid SoreThroat** | 0.061 | 0.015 | 0.042 | -0.020 | 0.074 | 0.023 | 0.024 | -0.006 | 0.033 |
| **Covid Vertigo** | 0.036 | 0.020 | 0.013 | 0.059 | -0.033 | -0.003 | 0.007 | 0.026 | 0.025 |
| **Covid hospitalization** | 0.030 | 0.004 | 0.006 | 0.063 | 0.038 | -0.003 | 0.006 | 0.005 | 0.019 |
| **BMI** | -0.229 | -0.016 | 0.006 | -0.261 | -0.185 | 0.007 | 0.005 | -0.044 | 0.094 |

| **Moderna D2** | **Dose2_Fever** | **Dose2_Fatigue** | **Dose2_Headache** | **Dose2_Nausea** | **Dose2_Chills** | **Dose2_Joint_Pain** | **Dose2_Muscle_Pain** | **Dose2_ Local side effects** | **Avg** |
| --- | --- | --- | --- | --- | --- | --- | --- | --- | --- |
| **Sex** | 0.357 | 0.023 | 0.318 | -0.847 | 0.119 | 0.532 | 1.007 | -0.322 | 0.441 |
| **Age** | -1.019 | -0.492 | -0.643 | -3.514 | -1.850 | -1.350 | -1.832 | -0.092 | 1.349 |
| **A** | -0.485 | 0.000 | -0.102 | -1.184 | -0.572 | -0.567 | -0.391 | -1.150 | 0.557 |
| **AB** | 0.947 | -0.034 | -0.401 | -1.683 | -1.301 | 0.635 | 1.559 | 0.143 | 0.838 |
| **B** | -0.094 | 0.472 | 0.478 | -0.335 | -0.840 | -0.725 | 0.186 | 0.666 | 0.474 |
| **O** | 0.194 | 0.147 | 0.186 | -1.006 | -0.573 | -1.177 | -0.304 | -0.059 | 0.456 |
| **Smoking** | -0.003 | -0.065 | -0.142 | -0.128 | -0.669 | -1.125 | -0.854 | -0.685 | 0.459 |
| **Substance use** | 0.000 | 0.000 | 0.000 | 0.000 | 0.000 | 0.000 | 0.000 | 0.000 | 0.000 |
| **Alcohol dependancy** | 0.577 | 0.206 | -0.179 | 1.662 | 1.650 | 2.066 | 1.451 | 0.299 | 1.011 |
| **Background Diabetes** | 0.235 | 0.042 | 0.075 | 0.407 | 1.626 | 1.408 | -0.054 | 0.361 | 0.526 |
| **Background Cardiovascular disease** | 0.576 | -0.465 | -0.810 | -0.557 | -0.223 | -0.957 | -2.678 | 0.153 | 0.802 |
| **Background Hypertension** | -1.465 | -0.300 | -0.897 | -2.304 | -1.625 | -2.020 | -1.882 | -0.180 | 1.334 |
| **Background CancerPassive** | 0.000 | 0.000 | 0.000 | 0.000 | 0.000 | 0.000 | 0.000 | 0.000 | 0.000 |
| **Background CancerActive** | 0.000 | 0.000 | 0.000 | 0.000 | 0.000 | 0.000 | 0.000 | 0.000 | 0.000 |
| **Background Neurological** | 0.407 | 0.012 | -0.211 | 1.783 | 0.177 | -1.329 | -1.828 | -0.010 | 0.720 |
| **Background pulmonary** | 0.097 | 0.118 | 0.206 | -2.506 | -1.002 | -0.438 | -0.150 | -0.287 | 0.601 |
| **Background ImmuneSystem** | -0.477 | -0.099 | -0.830 | -2.464 | 0.146 | -1.340 | -1.033 | 0.267 | 0.832 |
| **Background hematologic** | -0.309 | -0.284 | -0.280 | -0.123 | -0.521 | -0.152 | -0.962 | 0.119 | 0.344 |
| **Background Gastrointestinal** | 0.673 | 0.393 | 0.260 | 1.488 | -1.215 | 2.409 | 0.084 | 0.015 | 0.817 |
| **Background Renal** | 0.227 | -0.264 | -0.548 | -0.488 | 1.547 | -0.796 | -1.730 | 0.067 | 0.708 |
| **Background Hepatic** | 0.544 | 0.160 | 0.447 | 2.097 | 1.701 | -0.732 | 0.996 | 0.231 | 0.864 |
| **Background Skeletal** | 0.000 | 0.000 | 0.000 | 0.000 | 0.000 | 0.000 | 0.000 | 0.000 | 0.000 |
| **Background Mental** | -0.521 | -0.274 | -0.840 | -3.743 | -2.524 | -4.308 | -2.621 | -0.297 | 1.891 |
| **Background Allergy** | -0.115 | 0.445 | 0.038 | -1.642 | -0.061 | 0.134 | -1.639 | -0.961 | 0.629 |
| **Background None** | 0.212 | 0.224 | -0.439 | -0.933 | -0.185 | -0.086 | -1.679 | 0.229 | 0.498 |
| **Hormonal med** | 0.135 | 0.680 | -0.098 | 0.081 | 0.321 | 0.696 | 0.126 | 0.038 | 0.272 |
| **Pregnancy** | 0.335 | 0.357 | 0.562 | 1.663 | 0.753 | 1.650 | 1.172 | -0.134 | 0.828 |
| **Respiratory Inhaler** | -0.325 | -0.109 | -0.168 | 0.108 | -0.648 | -0.775 | -0.202 | -0.366 | 0.338 |
| **Corticosteroid Med** | -0.797 | 0.257 | -1.326 | -0.831 | -1.007 | -2.495 | 0.065 | -0.646 | 0.928 |
| **Chemotherapy Med** | 0.475 | 0.099 | 1.096 | 1.097 | 3.196 | 1.588 | 2.189 | 0.338 | 1.260 |
| **Immunosuppressive Med** | -0.566 | -0.204 | 0.086 | -0.490 | -1.508 | 1.552 | -3.428 | 0.458 | 1.036 |
| **Covid Infection** | -0.713 | -0.142 | -0.125 | -0.810 | -1.552 | -1.305 | -2.104 | 0.289 | 0.880 |
| **Covid Fever** | -0.096 | -0.200 | -0.214 | -0.165 | -0.113 | -0.368 | -0.184 | 0.234 | 0.197 |
| **Covid Fatigue** | -0.096 | -0.200 | -0.214 | -0.165 | -0.113 | -0.368 | -0.184 | 0.234 | 0.197 |
| **Covid Cough** | 0.006 | 0.003 | -0.005 | 1.106 | 0.124 | -0.832 | -0.528 | 0.188 | 0.349 |
| **Covid Gastrointestinal** | -0.102 | -0.202 | -0.210 | -1.271 | -0.236 | 0.464 | 0.345 | 0.046 | 0.359 |
| **Covid Anosmia** | -0.027 | -0.230 | 0.338 | 0.677 | 0.428 | -0.044 | -0.650 | 0.276 | 0.334 |
| **Covid RespiratoryProblems** | -0.228 | 0.183 | 0.203 | -1.751 | -0.373 | 0.784 | 0.760 | 0.051 | 0.542 |
| **Covid ConsciousnessDisorder** | 0.000 | 0.000 | 0.000 | 0.000 | 0.000 | 0.000 | 0.000 | 0.000 | 0.000 |
| **Covid Paresis** | 0.000 | 0.000 | 0.000 | 0.000 | 0.000 | 0.000 | 0.000 | 0.000 | 0.000 |
| **Covid ChestPain** | 0.722 | 0.381 | 0.861 | 1.083 | 2.234 | 1.211 | 1.745 | 0.111 | 1.044 |
| **Covid Headache** | -0.096 | -0.200 | -0.214 | -0.165 | -0.113 | -0.368 | -0.184 | 0.234 | 0.197 |
| **Covid SoreThroat** | 0.006 | 0.003 | -0.005 | 1.106 | 0.124 | -0.832 | -0.528 | 0.188 | 0.349 |
| **Covid Vertigo** | 0.746 | 0.100 | 0.329 | 0.217 | 1.962 | 2.465 | 2.225 | 0.044 | 1.011 |
| **Covid hospitalization** | 0.000 | 0.000 | 0.000 | 0.000 | 0.000 | 0.000 | 0.000 | 0.000 | 0.000 |
| **Dose1_Fever** | 1.683 | -0.170 | 0.117 | 0.390 | 0.311 | 0.880 | 0.091 | 0.228 | 0.484 |
| **Dose1_Fatigue** | 0.360 | 1.238 | 0.528 | 0.524 | 0.182 | 0.801 | 0.173 | 0.577 | 0.548 |
| **Dose1_Headache** | -0.439 | -0.229 | 0.656 | -0.056 | -0.186 | -0.034 | 0.307 | 0.222 | 0.266 |
| **Dose1_Nausea** | 0.099 | 0.145 | -0.020 | 1.653 | -1.280 | 0.262 | -0.168 | -0.054 | 0.460 |
| **Dose1_Chills** | 0.337 | -0.071 | -0.304 | -0.997 | 1.001 | -1.056 | -0.652 | -0.342 | 0.595 |
| **Dose1_Joint_Pain** | 0.030 | 0.020 | -0.714 | 0.356 | -0.255 | 2.072 | -0.074 | -0.486 | 0.501 |
| **Dose1_Muscle_Pain** | -0.613 | -0.181 | -0.446 | -0.118 | 0.274 | -0.458 | 1.434 | -0.004 | 0.441 |
| **Dose1_ Local side effects** | -0.120 | 0.010 | 0.201 | 1.640 | 0.551 | -0.302 | 0.815 | 2.421 | 0.757 |
| **BMI** | -0.063 | 0.385 | -1.378 | -0.892 | -0.339 | 1.209 | -0.435 | 0.031 | 0.592 |
